# Fatty acid-binding protein 5 aggravates psoriasis and psoriasis-like disease through ferroptosis

**DOI:** 10.1038/s41418-025-01630-4

**Published:** 2025-12-06

**Authors:** Kamil Mieczkowski, Latifa Bakiri, Bruna S. Martins, Kazuhiko Matsuoka, Erwin F. Wagner

**Affiliations:** 1 Department of Laboratory Medicine, Laboratory Genes and Disease, Medical University of Vienna (MUW), Vienna, Austria; 2 Department of Laboratory Medicine, Medical University of Vienna (MUW), Vienna, Austria; 3 Laboratory Bone Cancer Metastasis, Cellular and Molecular Tumor Biology, Center for Cancer Research, Medical University of Vienna (MUW), Vienna, Austria; 4 Department of Dermatology, Laboratory Genes and Disease, Medical University of Vienna (MUW), Vienna, Austria

**Keywords:** Chronic inflammation, Immunological disorders

## Abstract

Psoriasis (Ps) is a chronic inflammatory skin disease with systemic manifestations, such as psoriatic arthritis (PsA), cardiovascular and psychiatric complications, and subsequent negative effects on patients’ quality of life. Although biologics targeting specific disease mediators have become a mainstay in Ps treatment, exploration of new disease targets to improve treatment is still needed. Here we show that fatty-acid binding protein 5 (Fabp5) promotes skin inflammation through a therapeutically relevant modulation of the ferroptotic response. In epidermal-specific inducible c-Jun and JunB knockout (DKO*) mice, a preclinical model for Ps with PsA-like manifestations, dermal fat is reduced, serum free fatty acids (FFA) decreased, and β-hydroxybutyric acids (β-OHB) altered. Comparing RNA-seq and proteomic datasets from DKO* mice and Ps patients revealed shared alterations in fatty acid metabolism and ferroptosis signatures. Specifically, increased expression of Fabp5 and decreased expression of glutathione peroxidase 4 (Gpx4), a lipid-modifying enzyme and ferroptosis suppressor, are observed in the epidermis of DKO* mice and Ps patients. Treatment of DKO* mice with the Fabp inhibitor BMS increased Gpx4 expression, reduced lipid peroxidation products and neutrophil infiltration, ameliorated the skin phenotype, and alleviated keratinocyte hyperproliferation without affecting systemic IL-17a signaling and PsA-like manifestations. Importantly, dysregulated epidermal Fabp5 and Gpx4 expression was normalized after anti-IL17a or anti-TNFα antibody administration in DKO* mice, as well as in Ps patients treated with the corresponding Ps biologics. Furthermore, treatment with the ferroptosis inhibitor, liproxstatin-1, suppressed Ps-like skin thickening in DKO* mice, but did not affect the joint phenotype. These results support a functional and disease-relevant link between Fabp5, Gpx4 and ferroptosis in the skin that should be therapeutically exploited.

## Introduction

Psoriasis (Ps) is a chronic inflammatory, systemic disorder with a global prevalence of 0.3–1% [1–3]. The uncontrolled systemic inflammatory response contributes to a number of Ps comorbidities, including psoriatic arthritis (PsA), cardiometabolic disease and diverse aspects of the metabolic syndrome, such as obesity, fatty liver disease, hypertension, dyslipidemia, and diabetes [4–9]. Ps affects both the physiological and mental functioning of patients, leading to a reduced quality of life [10–12]. Genetic factors and environmental stimuli, such as obesity, metabolic factors, infections, skin injury, smoking, and stress, are involved in the etiology of Ps [13–18]. Both keratinocytes (KCs) and cells of the innate and adaptive immune system, such as dendritic cells, neutrophils and T cells, contribute to Ps pathogenesis. A dysregulated cross-talk between these cells within the skin leads to secretion of proinflammatory cytokines, alarmins and chemokines, such as TNFα, IL-17, IL-23, IL-6, IL-1β, S100A8/A9 and CXCL1/3/5, that spread cutaneous inflammation to the whole organism [19].

Recent studies suggested that a shift in the redox balance towards oxidative conditions with increased reactive oxygen species (ROS) production is associated with Ps progression [20]. ROS-dependent lipid peroxidation products, such as 4-hydroxynonenal (4-HNE), an aldehyde product of phospholipid fragmentation, and 8-isoprostane, the product of phospholipid cyclization, are increased in KCs of Ps patients [21]. 4-HNE and 4-HNE-protein adducts are also significantly higher in the plasma and in blood cells of Ps and PsA patients [22, 23]. On the other hand, the activity of antioxidant enzymes, including glutathione peroxidase, glutathione reductase and thioredoxin reductase, are decreased in the plasma of Ps patients [23]. Furthermore, skin samples from severe Ps patients show increased lipid peroxidation, decreased superoxide dismutase and catalase activity, and a more compromised total antioxidant status compared to those from patients with mild to moderate Ps [24]. However, the impact of dysregulated lipid peroxidation in Ps pathogenesis, as well as direct comparisons between clinical forms of the disease, remain underexplored.

The lipid chaperone fatty acid-binding protein 5 (Fabp5) is involved in lipid-mediated transcriptional regulation, lipid droplet storage, signal transduction, mitochondrial and peroxisomal oxidation and cell death [25]. Fabp5 binds long-chain fatty acids, such as arachidonic acid, facilitates their distribution to several intracellular compartments, including the nucleus, where these serve as ligands for transcription factors, such as Ppars [26]. Fabp5 is increased in the skin and the serum of Ps patients, and KC-expressed Fabp5 promotes murine skin inflammation through cytokine-mediated neutrophil recruitment [27]. Fabp5 is also upregulated during ferroptosis, a form of cell death induced by lipid peroxidation and iron overload, where it promotes the redistribution of redox-sensitive lipids and ferroptosis sensitivity in a positive-feedback loop [28]. Ferroptosis, characterized by a decrease in the antioxidant enzyme and ferroptosis inhibitor Gpx4 and an increase in proteins containing 4-HNE adducts, was observed in the skin of Ps patients [29, 30]. Inhibition of lipid peroxidation alleviated IMQ-induced skin inflammation [29], while KC-specific *Gpx4* gene inactivation in a subset of basal KC was recently shown to initiate a ferroptosis program in these cells, leading to Ps-like features in mice [30]. Fabp5, lipid peroxidation and possibly Gpx4/ferroptosis could therefore be functionally connected during Ps progression and relevant for prognosis and therapy, but to date, this link has not been evaluated.

Genetically-engineered mouse models (GEMMs) have been generated to model Ps [31–37]. The tamoxifen (TAM)-inducible, epidermal-specific, c-Jun and JunB double knockout (DKO*) develops several psoriatic hallmarks, including PsA-like arthritic joints [38, 39]. Hypothesis-driven analyses as well as preclinical studies in the DKO* mice validated several Ps/PsA mediators and therapeutic targets, such as TNFR1 signaling [35], the S100A8/S100A9 alarmin [39, 40], complement C3 [40], VEGF [41], IL-23 [42] and TSLP [38]. Contribution of some of these factors in Ps pathogenesis was also demonstrated by other GEMMs such as epidermal-specific IL-23 overexpression (R23) [37] and gain-of-function CARD14 (CARD14^E138A^) [34] models. Although different drivers initiate Ps-like skin inflammation, these mouse models show similar skin phenotypes, including KCs hyperproliferation, aberrant KCs differentiation, skin barrier disruption and increased immune cell infiltration and blocking IL-23/IL-17 axis restores these skin phenotypes [33, 34, 36].

Using the DKO* GEMM, preclinical therapeutic approaches as well as murine and human datasets, we show for the first time that competitive inhibition of Fabp5 binding to endogenous fatty acids modulates Gpx4 levels, lipid peroxidation and ferroptosis-associated gene expression downstream of TNFα/IL-17a-driven, Ps-like skin inflammation. Thus, targeting Fabp5-dependent lipid peroxidation and/or suppression of ferroptosis through Gpx4 could be therapeutically relevant to efforts aiming at potentiating current Ps treatments.

## Results

### Psoriasis-like disease affects dermal and peripheral adipose tissue

**Fig. 1:**
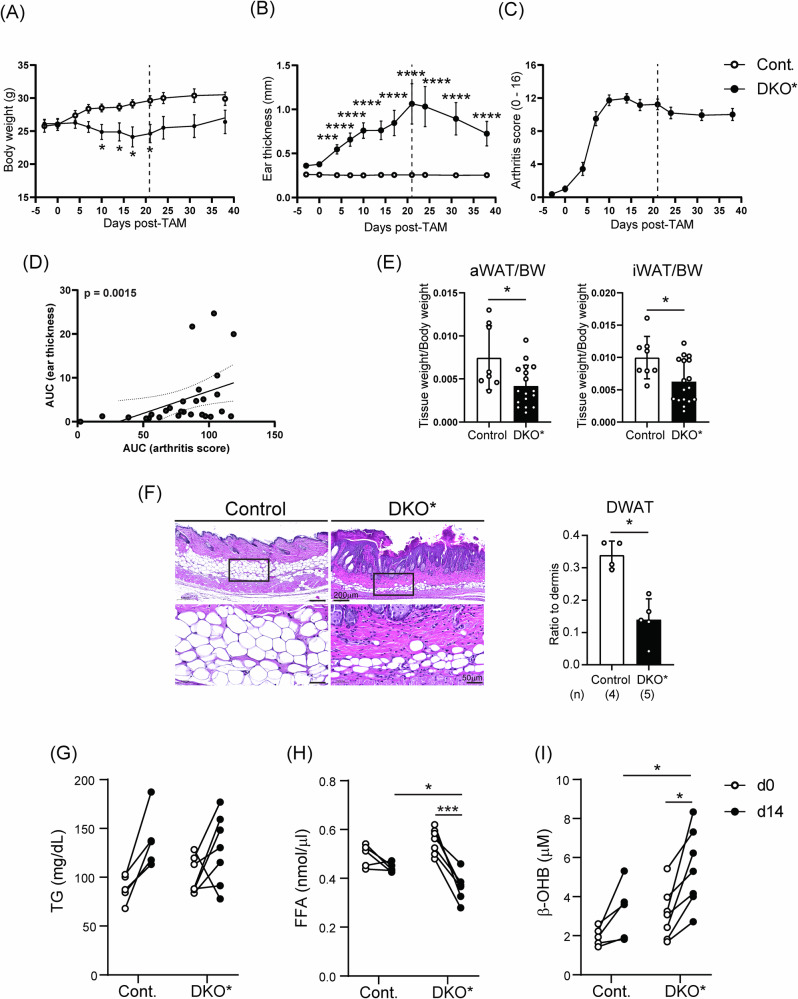
Epidermal c-Jun/JunB deletion-driven skin/systemic inflammation reduces body weight and adipose tissue mass. **A** Body weight. **B** Ear thickness. **C** Arthritis score. **D** Correlations of area under the curve (AUC) for ear thickness and arthritis score in DKO* mice. **A–C** Bar graphs and plots represent or include mean ± s.e.m, respectively. **E** Quantification of axillary and inguinal adipose tissue weight at endpoint (38 days post-TAM). Statistical differences between groups were analyzed by the Mann-Whitney test. **F** Representative images of H&E staining of back skin (left) and histological quantification of the dermal white adipose tissue (DWAT) area (right). Statistical differences between groups were analyzed by the Mann-Whitney test. **G–I** Serum levels of TG, FFA and β-OHB before (d0: before TAM injection) and after TAM (d14: 14 days post-TAM injection). **G** TG. **H** FFA. **I** β-OHB. Statistical differences between groups were analyzed by two-way ANOVA with Bonferroni post hoc analysis. *P < 0.05, **P < 0.01, ***P < 0.001.

DKO* mice were injected with TAM at 8 weeks of age to induce psoriasis-like disease, and mice were monitored for body weight (BW), ear thickness and arthritis severity over a time course of 38 days (Fig. 1A–D, Supplementary Fig. 1A–D). BW loss started 10 days post-TAM injection, recovered around 20 days and was largely similar to control littermates at the endpoint (Fig. 1A and Supplementary Fig. 1B). Ear thickness and joint arthritis scores began to increase 5 days after TAM injection, peaking at around day 20 (Fig. 1B, C). Interestingly, while arthritis persisted, ear skin thickness started to decrease after 20 days, suggesting distinct lesion dynamics between skin and joints. Qualitative histological analyses at the endpoint, 38 weeks post-TAM injection, revealed that cortical phalangeal bones in DKO* mice with high arthritis scores were thinner than those in mice with lower scores or in control mice (Supplementry Fig. 1D). Nevertheless, ear thickness in DKO* mice remained higher than in controls (Fig. 1B) and the area under the curves (AUC) of ear thickness and arthritis scores correlated positively between day 0 and the endpoint (Fig. 1D). DKO* mice displayed higher spleen and liver weights and lower axillary/inguinal white adipose tissues (a/iWAT) weights relative to BW at the endpoint, while brown adipose tissue (BAT) and skeletal muscle were not altered (Fig. 1E and Supplementry Fig. 1E). Dermal white adipose tissue (DWAT) was also strikingly reduced in the back skin of DKO* mice compared to controls (Fig. 1F). Furthermore, circulating FFA decreased in DKO* mice between day 0 and day 14 and their catabolic product, β-OHB, increased, while triglycerides (TG) were unchanged, and none of these parameters were affected in controls (Fig. 1G–I). These findings indicate that the Ps-like disease in DKO* mice affects the adipose tissue and is associated with altered local and systemic lipid metabolism.

### Fabp5 and Gpx4 are dysregulated in the skin of DKO* mice, resembling Ps skin samples

**Fig. 2:**
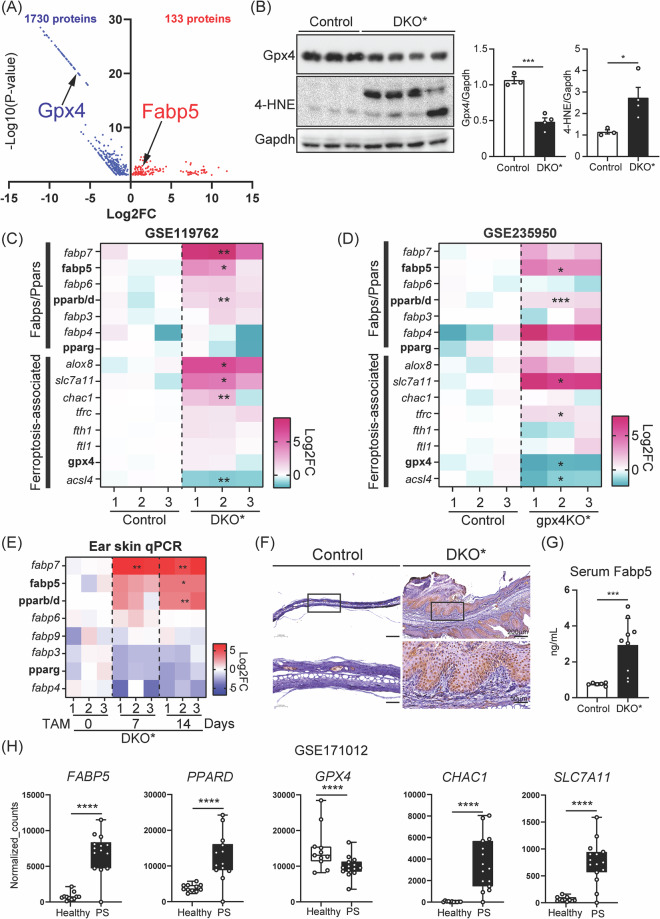
Dysregulation of fatty acid binding protein 5 and glutathione peroxidase 4 in DKO* mice. **A** Volcano plot showing upregulated (red) and downregulated (blue) proteins from 1863 differentially expressed proteins (DEPs) in whole ear skin lysate of DKO* mice compared to control mice (*n* = 3–5 per condition; *p* < 0.05). **B** Western blotting of Gpx4,4-HNE, and Gapdh using whole ear skin lysate from DKO* mice 4 weeks post-TAM injection (left) and the ratio of protein band density of Gpx4/Gapdh and 4-HNE/Gapdh (right). **C** Log2FC-based relative mRNA expression heat map of bulk RNA-seq of FACS-isolated basal keratinocytes from DKO* mice and control mice 7 days after Tamoxifen (GSE119762) [38]. **D** Log2FC-based relative mRNA expression heat map of bulk RNA-seq of whole epidermial skin from mice with inducible gene inactivation of Gpx4 in a subset of Keratin 14-expressing keratinocytes and controls 15–18 days after Tamoxifen injection (GSE235950) [30]. **E** Log2FC-based relative mRNA expression heat map of qRT-PCR using ears at different time-point post-TAM injection. **F** Representative IHC images of Fabp5 in the ear of control and DKO* mice 14 days post-TAM injection. **G** Serum Fabp5 in control and DKO* mice 14 days post-TAM injection (ELISA). **H** Selected transcriptomic changes in whole lesional skin tissue (bulk RNA-seq) from Ps patients compared to healthy individuals (GSE171012, before Secukinumab treatment [35]). Bar graphs and plots represent or include mean ± s.d., respectively. Statistical differences between groups were analyzed by the Mann-Whitney test. *P < 0.05, **P < 0.01, ***P < 0.001.

The lipid chaperone fatty acid-binding protein 5 (FABP5/Fabp5) was among the proteins prominently altered in proteomic analyses of lesional skin samples from Ps patients and DKO* mice, along with other key proteins contributing to lipid/arachidonic acid metabolism [40]. Fabp5 was also increased in an independent proteomic dataset generated from DKO* skin lysates [39] (Fig. 2A). However, Gpx4, a ferroptosis suppressor that neutralizes lipid hydroperoxides, was decreased in DKO* mutants in the same dataset (Fig. 2A). Reverse transcription followed by quantitative PCR (qRT-PCR) confirmed decreased *gpx4* mRNA levels in the skin of DKO* mice and further revealed dysregulated expression of ferroptosis-associated genes, such as arachidonate 8-lipoxygenase (*alox8*), acyl-CoA synthetase long chain family member 4 (*acsl4*), glutathione specific gamma-glutamylcyclotransferase 1 (*chac1*) and the glutamate/cystine antiporter (*slc7a11*) (Supplementry Fig. 2A). Decreased Gpx4 protein expression in DKO* mice was also apparent in Western blotting (WB) of whole ear skin lysates (Fig. 2B). Immunohistochemistry (IHC) indicated that while Gpx4 was rather evenly expressed in all epidermal layers of control mice, it was only detectable in basal KCs of DKO* mice (Supplementry Fig 2B). 4-HNE is a highly reactive byproduct of lipid peroxidation, a chain reaction that can be interrupted by Gpx4 activity. In contrast to the expression pattern of Gpx4, IHC analysis showed that 4-HNE protein adducts were abundant in the upper epidermis of DKO* mice and less prominent in basal KCs (Supplementry Fig. 2B). Increased 4-HNE-protein adducts were also detected in DKO* whole ear skin lysates, indicative of increased lipid peroxidation and consistent with decreased Gpx4 activity, while terminal deoxynucleotidyl transferase dUTP nick end labeling (TUNEL) positive foci were also present in the epidermis of DKO* mice (Fig. 2B and Supplementary Fig. 2C). RNA-seq analysis of basal KCs isolated by FACS [38] indicated that the dysregulated expression of ferroptosis-associated genes, such as *alox8, acsl4, chac1* and *slc7a11*, and increased expression of *fabp5*, *fabp7* and *pparb/d*, was already occurring 7 days after TAM injection in DKO* basal KCs (Fig. 2C). Interestingly, a similar Fabp/Pparg and ferroptosis gene expression profile was observed in the epidermis of TAM-inducible Gpx4 cKO* mice [30], where Gpx4 is inactivated in a subset of basal KCs (Fig. 2D). qRT-PCR analyses confirmed increased *fabp5* expression in DKO* whole ear skin extracts in comparison to control ear skin within a 2-week time course, along with *fabp7* and *pparb/d* (Fig. 2E and Supplementary Fig. 2D). Conversely, *pparg* was decreased in DKO* mice, and no difference in *fabp4* was detected between groups (Fig. 2E and Supplementary Fig. 2D). IHC further revealed that DKO* KCs expressing Fabp5 were localized in the basal and spinous layers of the epidermis, while the protein was only detected in the sebaceous glands of control mice (Fig. 2F). Increased serum Fabp5 was also measured in DKO* mice (Fig. 2G), consistent with the reported increase of circulating FABP5 in Ps patients [43]. In contrast, while IHC revealed patchy Fabp7 expression in basal DKO* epidermis, circulating Fabp7 was similar between DKO* and control mice (Supplementary Fig. 2E, F), and Fabp4 was only detected in the DWAT of both groups (Supplementary Fig. 2G). Interrogating publicly available human transcriptomic data sets revealed that *FABP5*, *PPARD*, *CHAC1* and *SLC7A11* were increased and *GPX4* decreased in skin samples of Ps patients compared to healthy individuals (Fig. 2H), while *FABP7, FABP4, PPARG, ACSL4, ALOX15B*, the human ortholog of *alox8*, were unchanged (Supplementary Fig. 2H). Mining of 6 additional transcriptomic data sets, where lesional and non-lesional skin samples from the same Ps patients were profiled, revealed increased *FABP5, CHAC1*, *SLC7A11* and decreased GPX4 expression in lesional skin samples (Supplementary Fig. 3). These results indicate that lipid metabolism and ferroptosis are dysregulated in DKO* mice and in Ps samples, and suggest that Fabp5 and Gpx4 may functionally contribute to disease pathogenesis by modulating these processes.

### Pharmacological targeting of FABP activity alleviates Ps-like skin disease

**Fig. 3:**
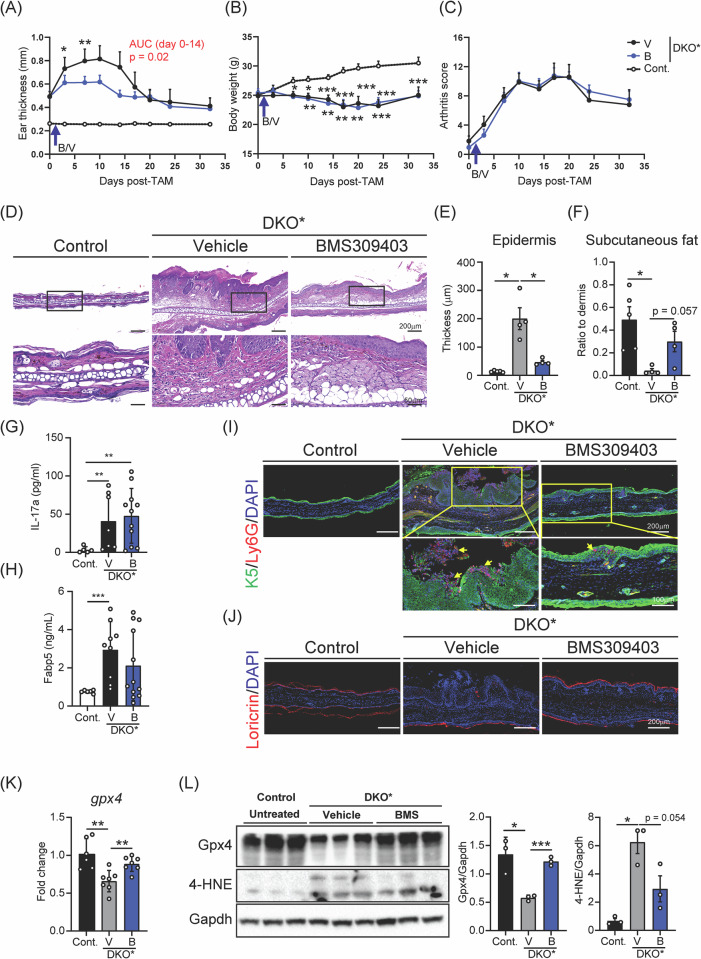
Systemic BMS309403 treatment reduces skin disease in DKO* mice. **A** Ear thickness. **B** Body weight. **C** Arthritis score. **D** Representative images of H&E staining of ears from DKO* mice treated with BMS309403/vehicle 14 days post-TAM. **E** Histological quantification of epidermal thickness 14 days post-TAM. **F** Histological quantification of DWAT area relative to the dermis. **G**, **H** ELISA using serum samples of BMS309403/vehicle-treated DKO* mice 14 days post-TAM injection. **G** IL-17a. **H** Fabp5. Representative immunofluorescence (IF) images of Keratin 5 (K5)/neutrophils (Ly6G) (**I**) and loricrin (**J**) in ear skin sections from control and BMS309403/vehicle-treated DKO* mice 14 days post-TAM injection. **K** RT-qPCR analysis of *gpx4* in ear skin extracts from controls and BMS309403/vehicle-treated DKO* mice 14 days post-TAM. **L** Western blotting of 4-HNE, Gpx4 and Gapdh using whole ear skin lysate from DKO* mice 14 days post-TAM. Blot quantifications are shown on the right. Bar graphs and plots represent or include mean ± s.d., respectively. Statistical differences between groups were analyzed by the Mann-Whitney test. *P < 0.05, **P < 0.01, ***P < 0.001.

Cohorts of DKO* mice were treated with BMS309403 (BMS), a potent inhibitor of Fabp3/4/5 activity (Supplementary Fig. 4A) [44, 45]. BMS treatment significantly suppressed skin thickening and reduced AUC in the ears of BMS-treated DKO* mice compared to vehicle-treated DKO* mice (Fig. 3A, D, E, Supplementary Fig. 4B). Histological analyses performed 14 days post-TAM confirmed the alleviation of epidermal thickening in BMS-treated mice (Fig. 3D, E), and no necrotic areas were observed histologically. DWAT (Fig. 3F) and dermal thickness (Supplementary Fig. 4C) were also restored to control levels. However, BMS treatment did not ameliorate BW loss, arthritis scores or spleen enlargement (Fig. 3B, C and Supplementary Fig. 4D). Notably, serum levels of IL-17a and Fabp5 remained unchanged between the BMS- and vehicle-treated groups (Fig. 3G, H). Histological assessment of ear sections 14 days post-TAM revealed decreased neutrophil infiltration (Ly6G + ), enhanced loricrin expression, and reduced Ki67 in BMS-treated DKO* mice (Fig. 3I, J and Supplementary Fig. 4E). qRT-PCR analysis at 14 days post-TAM indicated that BMS-treatment restored *gpx4* and *fabp7* expression, while ferroptosis-associated genes were not significantly changed (Fig. 3K and Supplementary Fig. 4F). WB analyses of whole ear skin lysates confirmed the recovery of Gpx4 expression in BMS-treated DKO* mice 14 days post-TAM, and revealed a reduction in 4-HNE-protein adducts (Fig. 3L). IHC of skin tissues further demonstrated expansion of the Gpx4-positive KCs into the upper epidermal layers, 4-HNE-positive KCs and TUNEL-positive foci were reduced (Supplementary Fig. 4G, H). These data indicate that Fabp activity contributes functionally to the Ps-like cutaneous but not systemic phenotypes of DKO* mice, likely acting downstream of IL-17a signaling and possibly upstream of Gpx4 expression and lipid peroxidation.

### Targeting Ps-relevant cytokines normalizes altered Fabp5 and Gpx4 expression in the skin of DKO* mice and Ps patients

**Fig. 4:**
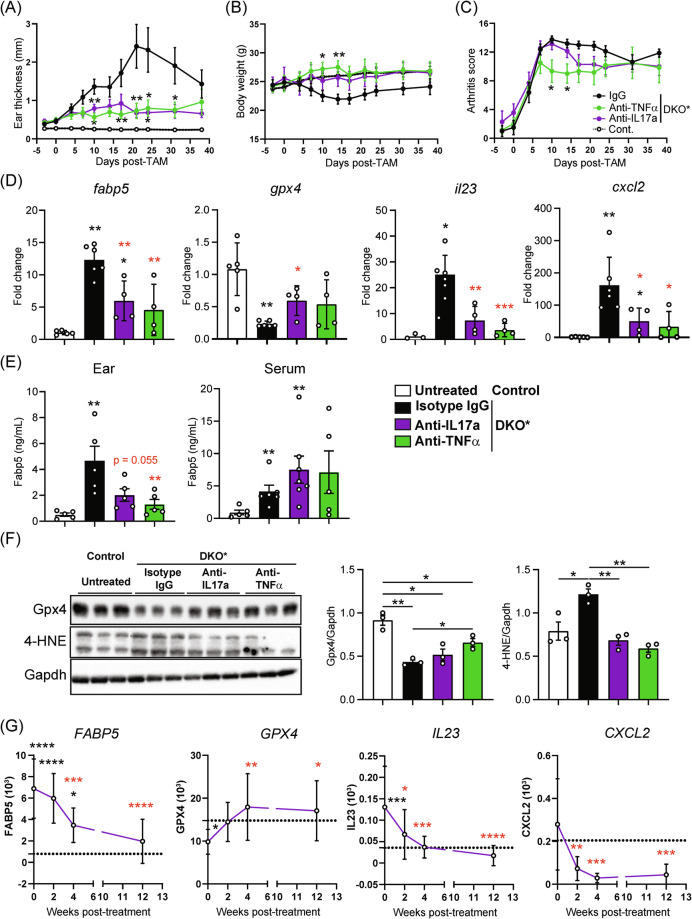
Targeting Ps-relevant cytokine pathways reverts Fabp5 and Gpx4 expression in DKO* mice and Ps patients. **A** Ear thickness. **B** Body weight. **C** Arthritis score. **A–C** Bar graphs and plots represent or include mean ± s.e.m, respectively. **D** RT-qPCR analysis of *fabp5, gpx4* and other Ps-related genes using the ear of DKO* mice treated with antibodies 38 days post-TAM injection. Black and red asterisks indicate comparisons between untreated control and DKO* mice and between isotype IgG and anti-IL17a/anti-TNFα antibodies, respectively. **E** Fabp5 ELISA using whole skin (ear) lysate (left) and serum (right) of Ab-treated DKO* mice 38 days post-TAM injection. **F** Western blotting of Gpx4, 4-HNE and Gapdh using whole ear skin lysate from DKO* mice 38 days post-TAM injection (left) and the ratio of protein band density of 4-HNE/Gapdh and Gpx4/Gapdh (right). **G** Selected longitudinal transcriptomic changes (bulk RNA-seq) in whole lesional skin tissue from Secukinumab-treated Ps patients (GSE171012) [35]. For each gene, the dashed line indicates the expression level measured in healthy individuals from the same dataset. D–F Bar graphs and plots represent or include mean ± s.d., respectively. Statistical differences between groups were analyzed by one-way ANOVA with Tukey’s multiple comparisons test. *P < 0.05, **P < 0.01, and ***P < 0.001. Black and red asterisks indicate comparisons between healthy individuals and Ps patients and between week 0 and each time point during secukinumab treatment, respectively.

Next, DKO* mice were treated with anti-IL17a or anti-TNFα antibodies (Abs) (Supplementary Fig. 5A). Both Ab-treated groups exhibited a significantly alleviated skin phenotype compared to the isotype IgG-treated group, both macroscopically throughout the treatment period (Fig. 4A) and confirmed histologically at the end-point, 38 days post-TAM injection (Supplementary Fig. 5B, C). However, anti-IL17a had no effect on BW loss and arthritis scores, and anti-TNFα treatment only transiently ameliorated these parameters (Fig. 4B, C). Dermal thickness, DWAT and peripheral fat deposits were also not affected by either Abs at the endpoint of the experiment (Supplementary Fig. 5C–E). Importantly, *fabp5* mRNA expression was reduced at the endpoint following both Ab treatments, along with several Ps/PsA-related inflammatory genes, including *il23, il1b, cxcl2, s100a8, s100a9* and, to a lesser extent, *il36g* (Fig. 4D and Supplementary Fig. 5F). Conversely, *gpx4* mRNA was increased and *slc7a11* decreased, although only reaching statistical significance in the anti-IL17a group, while *chac1* was not overtly changed (Fig. 4D and Supplementary Fig. 5F). Interestingly, while ELISA analyses confirmed decreased Fabp5 in ear skin lysates, a slight increase was measured in the serum at the endpoint (Fig. 4F). WB of whole ear skin lysates and IHC using skin sections further documented a decrease in 4-HNE-protein adducts in anti-TNFα Ab-treated DKO* mice, but to a lesser extent with anti-IL17a treatment, consistent with elevated Gpx4 protein expression (Fig. 4F and Supplementary Fig. 5G). Furthermore, a reduction in TUNEL-positive foci was also observed in the skin of anti-IL17a and anti-TNFα Ab-treated mice (Supplementary Fig. 5H).

To evaluate the clinical relevance of these findings, we analysed data sets from 4 different psoriasis clinical trials evaluating Ps-relevant drugs, namely anti-IL17RA monoclonal Ab/Brodalumab, TNFα inhibitor/Etanercept, anti-IL-23p19 monoclonal Ab/Guselkumab and PF06700841, a TYK2_JAK1 inhibitor. Baseline data confirmed the inverse expression of *FABP5* and *GPX4* in patient samples (Supplementary Fig. 6A, B). Importantly and in line with our findings using the DKO* model, treatment across all trials led to a consistent and striking decrease in *FABP5*, *SLC7A11* and *CHAC1*, alongside an increase in *GPX4* over time (Supplementary Fig. 6C, D). Furthermore, RNA-seq profiles from plaque psoriasis patients treated with anti-IL17a Ab/secukinumab [46] (GSE171012) revealed a significant downregulation of disease-associated cytokines, such as *IL23, CXCL2, IL1B, S100A8, S100A9, IL36G*, as well as ferroptosis-associated genes, like *SLC7A11* and *CHAC1*, during the course of treatment. Conversely, *GPX4* expression, which was lower in Ps patients at treatment start, was restored to the level measured in healthy individuals (Fig. 4G and Supplementary Fig. 5I).

**Fig. 5:**
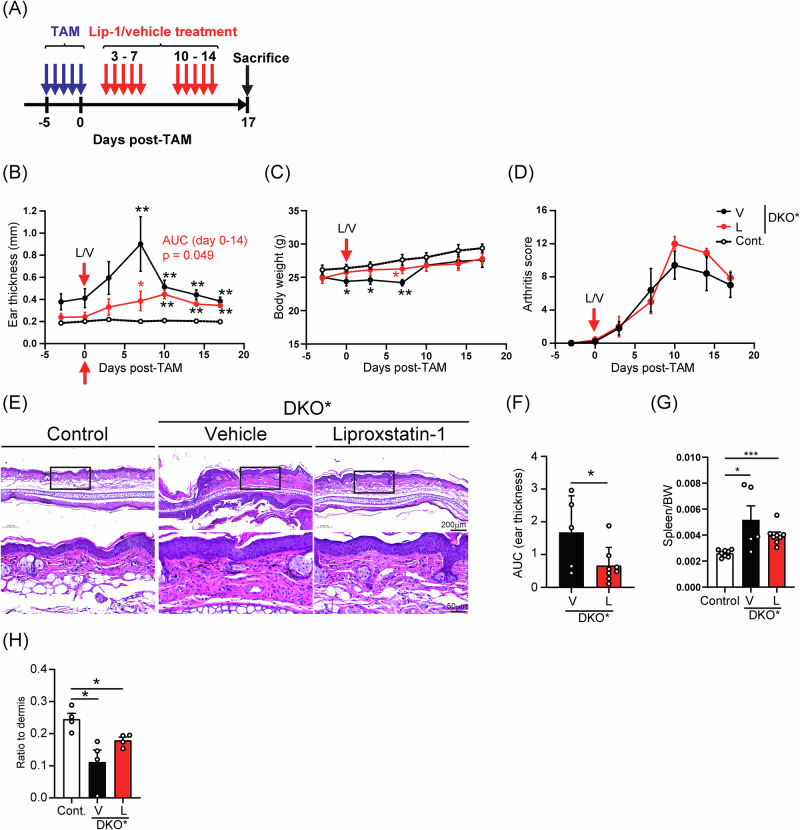
Systemic Liproxstatin-1 treatment reduces skin disease in DKO* mice. **A** Experimental procedure. Tamoxifen was injected into 8 week-old mice (2 mg/mouse/day, 5 consecutive days). Liproxstain-1/vehicle-treatment started 3 days after the last time TAM injection and lasted for 14 days (5 consecutive days/week). **B** Ear thickness. **C** Body weight. **D** Arthritis score. **E** Representative images of H&E staining of ears from DKO* mice treated with liproxstatin-1/vehicle 14 days post-TAM. **F** Quantification of AUC in Liproxstain-1/vehicle-treated DKO* mice between day 0 and day 14. **G** Quantification of spleen weight 14 days post-TAM injection. **H** Histological quantification of DWAT area relative to the dermis. Quantification of AUC in Liproxstain-1/vehicle-treated DKO* mice between day 0 and day 14. Bar graphs and plots represent or include mean ± s.d., respectively. Statistical differences between groups were analyzed by the Mann-Whitney test. *P < 0.05, **P < 0.01, ***P < 0.001.

To explore the therapeutic potential of targeting ferroptosis in skin inflammation, cohorts of DKO* mice were treated with Liproxstatin-1 (Lip-1), a potent ferroptosis inhibitor (Fig. 5A) [47]. Lip-1 treatment suppressed skin thickening and reduced AUC in the ears of DKO* mice compared to vehicle treatment, while no changes in body/spleen weight, arthritis score and DWAT were observed (Fig. 5B–H). Collectively, these data establish that both Fabp5 and Gpx4 are functionally implicated in Ps pathogenesis, and may serve both as biomarkers for treatment response and as therapeutic targets.

## Discussion

Despite the therapeutic success of biological agents blocking Th1/Th17, IL-23 and TNFα cytokines, the rate of non-responders remains high [48, 49] and their use is also limited by some adverse effects, such as infection, malignancy and cardiovascular risk [50]. It is therefore important to identify new targetable molecules/pathways that could be used either alone or in combination with Ps/PsA biologics.

The striking similarity between clinical data and mouse model indicates that FABP5 and GPX4 are associated with disease severity and pathogenesis, and both FABP5 and GPX4 could be used as biomarkers to assess the therapeutic effectiveness of biological agents. Since BMS treatment ameliorated skin inflammation and increased Gpx4 expression, but did not affect IL-17a levels in mice, Fabp5 and Gpx4 are therefore downstream of IL-23/IL-17a axis and/or TNFα signaling. We propose a new perspective on how these Ps-related inflammatory cytokines and downstream Fabp5 and Gpx4 changes are an important part of the inflammatory loop that amplifies skin inflammation. In the early stages of cutaneous inflammation in DKO* mice, inflammatory stimuli increase Fabp5 expression, which in turn modulates intracellular lipid metabolism through its fatty acid binding activity. This intracellular alteration may downregulate Gpx4 expression, resulting in increased lipid peroxidation, a shift in cellular redox balance toward oxidative conjugation, and induction of ferroptosis. This cascade may increase the production of cytokines, release of damage-associated molecular patterns through ferroptosis and promote further neutrophil infiltration, leading to amplification and establishment of Ps-like disease (Supplementary Fig. 7). Recent studies suggested that lipid peroxidation and ferroptosis, regulated by Fabp5 and Gpx4 independently, modulate T cell functions and anti-tumor immunity [51, 52]. Whether Fabp5 and Gpx4 might also modulate lipid peroxidation in DCs and/or neutrophils in Ps is worth exploring.

Since Fabp7 data was generally less prominent than Fabp5 data, making inter-species comparisons difficult, we focused on Fabp5. Furthermore, Fabp3 expression was below the qPCR detection limit, and Fabp4 was unchanged between control and DKO* whole skin KCs. In the dataset from Gxp4 KO skin, Fabp5 is locally and systemically increased. Therefore, we conclude that the predominant effect of the BMS treatment in DKO* mice occurs through Fabp5 inhibition. Since BMS, which competitively inhibits the lipid-binding activity of Fabp to endogenous fatty acid ligands, restored intracellular Gpx4 protein levels, the possible mechanisms may be as follows: Fabp5 channels polyunsaturated fatty acids into membrane phospholipids susceptible to peroxidation, promoting ferroptosis. Excessive accumulation of these peroxidation-prone phospholipids may overwhelm Gpx4 detoxification capacity via glutathione-dependent reduction and trigger a feedback loop that further impairs Gpx4 function [28, 53]. Alternatively, under oxidative stress, the nuclear factor erythroid 2-related factor 2 (NRF2) promotes *GPX4* transcription [54]. In parallel, Fabp5 may support this anti-/pro-oxidant response by delivering fatty acids to Pparβ/δ, enhancing its transcriptional activity and potentially influencing *GPX4* expression via Ppar-dependent mechanisms [55]. Interestingly, we recently described an anti-inflammatory role for p62, a well-characterized regulator of Keap1-Nrf2 complex, in Ps-like disease, using DKO* mice [56]. Whether p62 may be involved in the regulation of the Fabp5-Gpx4-ferroptosis axis needs to be further investigated.

A clinical report with a small sample cohort showed that cutaneous FABP5 protein level decreased in response to systemic TNFα inhibition prior to clinical improvement [57]. In accordance with this, we demonstrated that increased Fabp5 expression in DKO* skin was restored by inhibiting TNFα and IL-17 pathways, but circulating Fabp5 was not changed, and joint phenotypes remained unaffected, suggesting that local Fabp5 levels may reflect skin disease severity [57]. In mice, epidermal-specific Fabp5 deletion alleviated IMQ-induced skin inflammation through suppression of neutrophil infiltration [27], while SBFI-26, another Fabp5/7 inhibitor, ameliorated skin inflammation in the same model [58]. Although these studies seem to be consistent regarding the function of Fabp5, IMQ-induced skin inflammation, a model that exhibits strain- and sex-dependent variations, does not replicate any hallmarks of the human skin disease [59, 60]. Our study using a GEMM of Ps provides evidence that increased Fabp5 level and decreased Gpx4 level in keratinocytes are important contributors to Ps pathogenesis, and demonstrates for the first time that inhibition of Fabp activity and/or downstream lipid peroxidation could be beneficial for skin manifestations of Ps, but not for joint disease.

Epidermal-specific *Gpx4* gene inactivation in mice led to epidermal hyperplasia, an inflammatory infiltrate modulated in postnatal hair follicle morphogenesis, associated with elevated expression of cyclooxygenase-2 [61]. We found that in mutant mice, BMS treatment is beneficial in restoring Gpx4 expression to levels similar to wild-type mice with reduced 4-HNE-protein adducts, reversing ear skin lesions, but not PsA. Furthermore, systemic administration of liproxstain-1, which inhibits lipid peroxidation and ferroptosis, ameliorates the psoriasis-like phenotype in mice, suggesting that Gpx4-mediated ferroptosis suppression could be regulated by Fabp function and that Fabp-associated lipid metabolism can be involved in skin inflammation. Whether a Fabp5-Gpx4-ferroptosis cross-talk within the skin is cell-type specific remains to be further investigated.

Pparβ/δ is expressed in human and mouse KCs and increased in inflammatory conditions, such as Ps and wound healing, and influences keratinocyte proliferation and differentiation [62–66]. Topical application of Pparβ/δ agonist GW501516 on Pparβ/δ transgenic mice induced an inflammatory skin disease, and administration of anti-TNFα and IL-12 antibodies in mice reduced the skin phenotype [31]. It is therefore not surprising that in KCs, Fabp5 delivers endogenous ligands from the cytoplasm to nuclear Pparβ/δ to activate the cells, or that Ppar targets epidermal lipoxygenases (LOXs), such as ALOX15B/alox8 and ALOX12B/alox12b, and eicosanoids generated by LOXs then activate Pparβ/δ in a Fabp5-independent manner [64]. Fabp5 also contributes to lipid redistribution in cytosol and/or promotes mitochondrial damage through mediating lipid peroxidation in hypoxic/ischemic neurons [28, 67]. In line with this, lesional Ps skin contains abundant arachidonic acid and linoleic acid metabolites compared to non-lesional regions from healthy individuals [68, 69], implying that in Ps KCs, Fabp5 works with two arms: one is the Fabp5-Pparβ/δ axis, and the other is lipid peroxidation with Gpx4 suppression.

No effect on BW loss and PsA-like phenotype was observed for BMS, anti-IL17a and Lip-1 treatments, and only a transient amelioration was observed for anti-TNFα injection. The anti-TNFα data are consistent with our group’s previous study [35], demonstrating that genetic inhibition of TNF signaling (DKO* TNFR1 double knockout mice) improves the pathology of joint phenotypes. This suggests that more potent and/or complete inhibition of TNF-dependent pathways is necessary for the treatment of joint disease in DKO* mice. Clinical data indicate that anti-Ps biologics, including those targeting IL17A and TNFα, are more effective in improving psoriatic skin disease than in treating PsA [70–72], a phenomenon also observed in the DKO* model. Additionally, the etiology and progression of joint disease in psoriasis patients are more complex, often requiring higher doses of treatment [73].

Overall, this work highlights the involvement of Fabp5 in Ps pathogenesis and provides convincing evidence that systemic interventions, targeting Fabp proteins and ferroptosis, mimicking the effect of BMS/Lip-1 and/or preventing lipid peroxidation by restoring Gpx4 expression, might still be of interest to potentiate current therapies targeting skin manifestations in Ps. Further experiments are needed to determine whether restoring anti-oxidant properties combined with biologics can promote the therapeutic efficacy or reduce their dosage, and whether this combination strategy can reduce the risk of joint involvement and/or improve the inadequate response of current biologics.

## Materials and methods

### Genetically Engineered Mouse Models (GEMMs, phenotype monitoring and treatments

The generation of the DKO* psoriasis-like mouse model has previously been described [35]. Briefly, DKO* psoriasis-like mouse model with mixed background was generated by combining obtain *junB*^*f/f*^, *c-jun*^*f/f*^, and *K5-Cre-ERT* by genetic crosses. Eight-week-old male DKO* mice and controls (*junB*^*f/f*^*;c-jun*^*f/f*^) were injected daily (intraperitoneal), five times with 2 mg TAM (Sigma) and BW, ear thickness measured with a caliper and the extremities of the 4 limbs given an arthritis score every 2–3 days. Arthritis scores were determined on the extremities of each mouse, and the score for each mouse is the sum of the scores for all limbs (range: 0–16 points, with a maximum score in each mouse of 16). Scoring criteria were as follow: 0: healthy digit/paw. 1: One swollen digit. 2: Two swollen digits. 3: Three swollen digits. 4: All digits and paw are swollen. For antibody treatments, mice were randomized 3 days after the last TAM injection and weekly treated with anti-mouse IL-17a (150 μg/mouse/day) and anti-mouse TNFα (500μg/mouse/day) and isotype IgG (mouse IgG κ) (500 μg/mouse/day) antibodies by intraperitoneal injections for 5 weeks (total 5 injections). For BMS-309403 (BMS) treatment, 3 days after the last TAM injection, mice were randomized and daily treated with BMS (30 mg/kg/day) and/or vehicle (10% DMSO/90% corn oil) by oral gavage for 29 days. For Liproxstatin-1 (Lip-1; TargetMol Chemicals) treatment, mice were randomized 3 days after the last TAM injection and treated daily with Lip-1 (15 mg/mL) or vehicle (10% DMSO in PBS) by intraperitoneal injection for 2 weeks. BW, ear thickness and arthritis score were monitored. The sample size for each experiment was chosen based on well-established standards in the field for similar types of assays [35, 38–40]. The overall severity of the phenotype in DKO* mice was closely monitored based on our animal ethical approval, and no DKO* mice reached the human endpoint during treatment.

### Immunohistochemistry

Tissues, including ears, limbs, livers, axillary/inguinal white adipose tissues (a/iWAT) and muscles were dissected, and limbs were decalcified with 18% EDTA (pH 8.0) for 2 weeks before paraffin embedding for histology. Immunohistochemistry/immunofluorescence was performed on 5 μm thick sections. Slides were deparaffinized using xylene or citrus reagents and bathed in decreasing alcohol concentrations (100%-96%-70%-ethanol) followed by water washes. Deparaffinized tissue sections were treated with H_2_O_2_ for 30 min for IHC, and antigen retrieval was carried out using citrate buffer and a pressure cooker for 20 min. After permeabilization by 0.1% TritonX-100/PBS for 10 min, non-specific binding was blocked with 10% normal serum/PBS. Tissue sections were incubated with primary antibodies (Supplementary Table 2) overnight at 4 °C. Biotin/streptavidin amplification and HRP-based chromogen detection (VECTASTAIN ABC Kit or M.O.M. ® detection Kit, Vector Laboratories, Inc.) were used for IHC following the manufacturer´s instructions. IF sections were incubated with Alexa Fluor® 488 or 555 goat anti-rabbit IgG (H+L) for one hour, and nuclei were counterstained with DAPI. Images were recorded at an Olympus BX63 microscope. Image processing, measurements, assembly, and quantification were performed using ImageJ.

### RNA isolation and qPCR (RT-qPCR)

Total RNA was isolated using TRI reagent (Sigma-Aldrich), and complementary DNA was synthesized using Ready-To-Go-You-Prime-First-Strand Beads (GE Healthcare) or GoScript™ Reverse Transcription Mix, Oligo(dT) (Promega), and qPCR was performed using GoTaq qPCR Master Mix (Promega) and Eppendorf fluorescence thermocyclers, all according to the manufacturer’s instructions. The 2^ΔΔCT^ method was used to quantify the amplified fragments. Expression levels were normalized using at least one housekeeping gene. Primer sequences are listed in Supplementary Table 1.

### Bulk RNA-sequencing

Mouse: The preparation of RNA-seq libraries has previously been described (GSE119762) [38]. Human: The transcriptomic datasets (GSE171012, GSE34248, GSE41662, GSE41663, GSE53552, GSE136757 and GSE51440) were retrieved from the GEO database (http://www.ncbi.nlm.nih.gov/geo). Normalized counts from RNA-seq/DNA microarray on bulk skin tissue were plotted.

### Proteomic datasets analysis spectrometry

The generation of the proteomic dataset has previously been described [39]. Proteins containing one or more peptides with FDR ≤ 0.05 and *p* < 0.05 were extracted. Upregulated and downregulated peptides were displayed in red and blue, respectively.

### Protein isolation and Western blotting

Protein isolation and Western blotting have previously been described [56]. Briefly, ear and back skin samples were collected and snap-frozen in liquid nitrogen immediately after sacrificing the mice. Protein isolation for Western blotting and ELISA was prepared using equally sized parts of the ear and the back skin. Tissues were homogenized using ceramic beads in RIPA buffer (50 nM Tris-HCl, pH 8, 0.5% SDS, 150 mM NaCl, 1% Triton X-100, 0.5% sodium deoxycholate) supplemented with protease inhibitor cocktail tablets (Roche) and phosphatase inhibitor cocktail 2 (Sigma-Aldrich). Protein concentration was measured using the Pierce BCA Protein Assay Kit (Thermo Fisher Scientific, Waltham, MA, USA). Equal amounts of protein (30 µg) per lane were loaded, resolved in SDS-PAGE, and transferred onto a nitrocellulose membrane. The membranes were blocked in 5% skimmed milk in PBS-T and immunoblotted overnight with the following primary antibodies at 4 °C: Gpx4 (Abcam, EPNCIRI144, Cambridge, UK, dilution 1:1000), 4-HNE (Calbiochem, San Diego, CA, United States, dilution 1:500), Gapdh (HyTest Ltd., Turku, Finland, 5G4, dilution 1:5000). Next, after washing steps in PBS-T, appropriate secondary antibodies ECL^TM^ donkey anti-rabbit IgG-HRP (Amersham) or ECL^TM^ sheep anti-mouse IgG-HRP (Amersham) were used. Chemiluminescence detection was performed with Clarity^TM^ Western ECL Substrate (Bio-Rad) and acquired with a ChemiDoc Imager (Bio-Rad). Densitometry quantification of bands representing detected proteins was done with ImageJ software. Densitometry data are presented as mean relative values of detected protein to loading control (GAPDH) ratios from three lanes. The uncropped gel images of all western blots are shown in Supplementary Fig. 8.

### Biochemical analyses

Blood was collected in K3EDTA MiniCollect® tubes (Greiner Bio-One, Kremsmünster, Austria) by submandibular bleeding and plasma was obtained by centrifugation at 2000 × *g* for 15 min at 4 °C. Plasma triglyceride, free fatty acid and ketone body levels were measured according to manufacturer’s instructions using commercially available kits (GPO-PAP Triglyceride Liquicolor kit, HUMAN Biochemica and Diagnostica GmbH, Wiesbaden, Germany; Free Fatty Acid Assay Kit, Sigma-Aldrich, St Louis, Mo, USA; β-Hydroxybutyrate (Ketone Body) fluorometric Assay Kit, Cayman Chemical Company, Ann Arbor, Michigan, USA).

### Enzyme-Linked Immunosorbent Assay (ELISA)

Ear and skin protein lysates or sera were analysed using commercial ELISA kits according to the manufacturer’s instructions. The following ELISA kits were used: mouse Fabp5 (Novus Biologicals, NBP2-82411) and mouse IL-17a (R&D, DY421-05).

### Statistical analyses

Statistical analyses were performed using Prism (GraphPad 10 Software Inc., USA). Data are presented as mean ± standard deviation (SD) or ± standard error of the mean (SEM), as indicated in the figure legends. When comparing two groups, the Mann-Whitney *U* test was used, and to compare three or more groups, one-way ANOVA and Tukey’s method for multiple comparisons were implemented. For human gene expression profiling datasets, differences between two time-points during treatment (i.e., week 0 and weeks post-treatment) and between healthy individuals and patients were compared Student *t* test (paired) and the Mann-Whitney *U* test (unpaired), respectively. *P* < 0.05 was considered as statistically significant.

## Supplementary information

Supplementary figure and legends

Primers

Antibody

Original Data Files
