# Supplementary material for: Fatty acid-binding protein 5 aggravates psoriasis and psoriasis-like disease through ferroptosis
Source: Cell Death Differ. 2025 Dec 6;33(7):1333–44. doi: 10.1038/s41418-025-01630-4 (PMC13342077; doi:10.1038/s41418-025-01630-4)
Supplement: Supplementary file 1 — Supplementary figure and legends [file 41418_2025_1630_MOESM1_ESM.pdf]

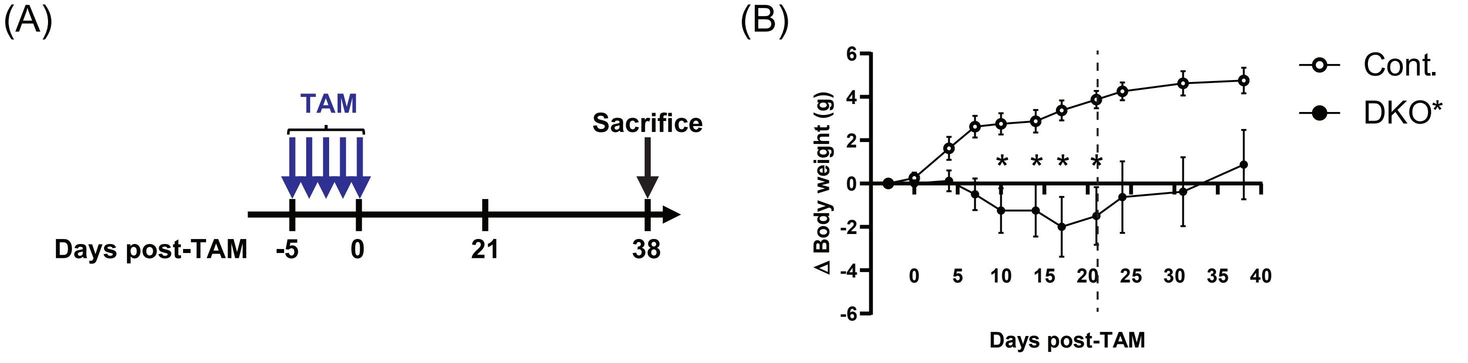

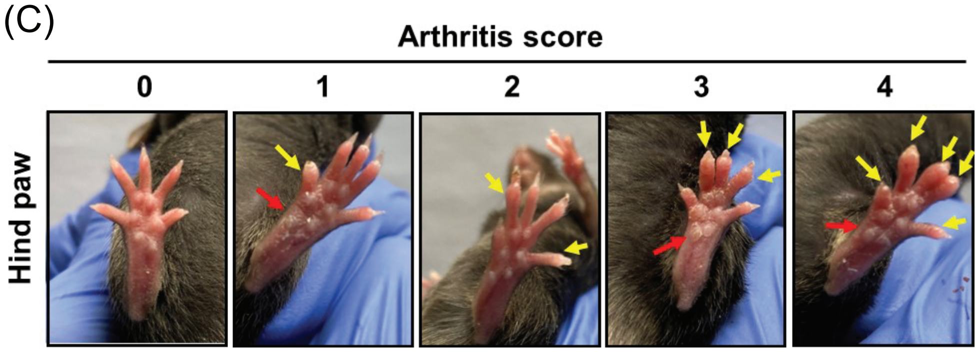

Score	Arthritis score (PsA-like lesions)
0	Healthy
1	One swollen digit
2	Two swollen digits
3	Three swollen digits
4	All digits and paws swollen

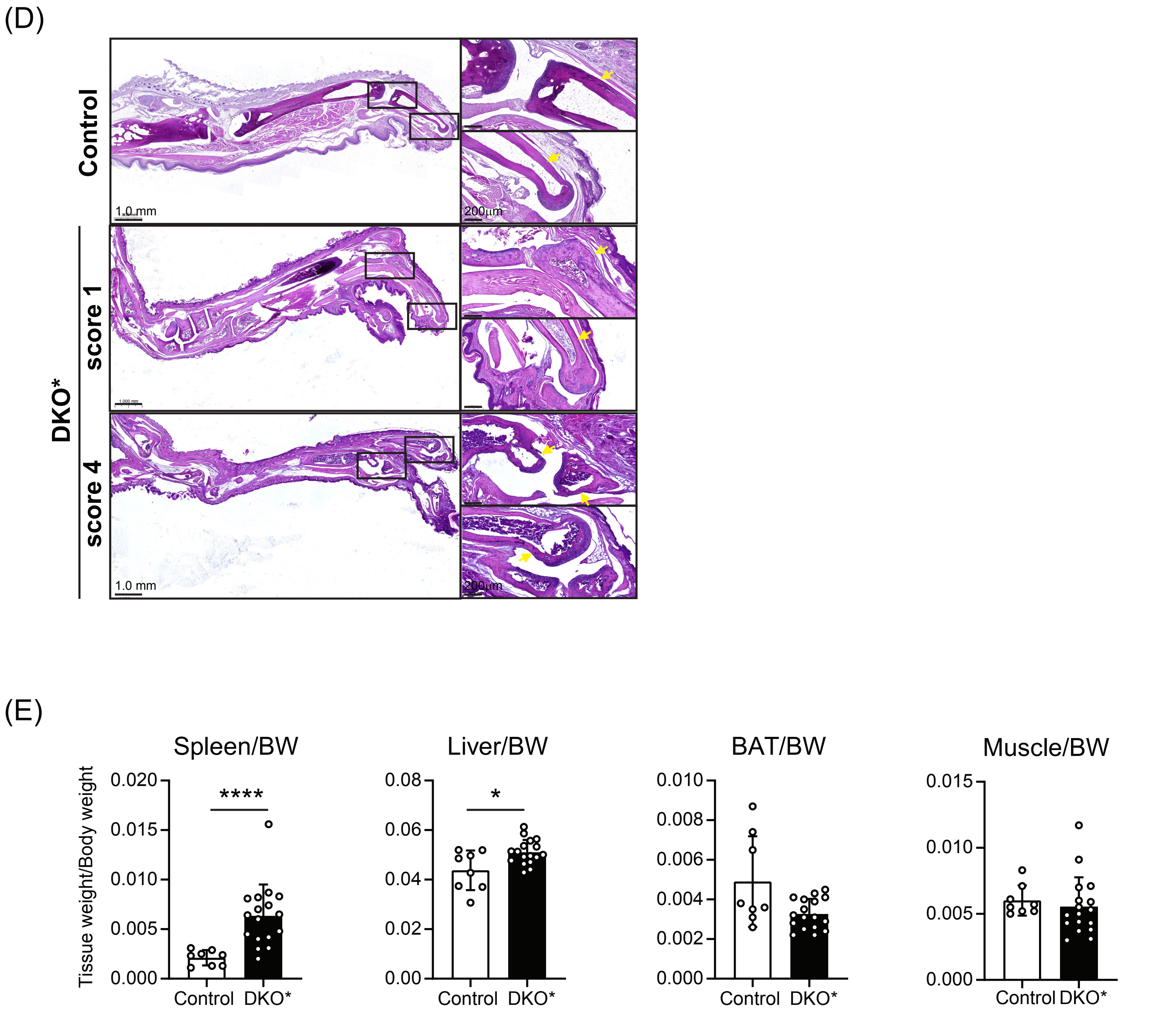

Supplemental figure legends

Supplemental Figure 1. Related to Figure 1.

(A) Experimental procedure and timeline to delete *c-jun and junb* in the epidermis. Tamoxifen was intraperitoneally injected into 8 weeks-old control and DKO* mice (2mg/mouse/day, 5 consecutive days) and body weight, ear thickness and arthritis score were monitored (day 0 = last injection). **(B)** ∆BW (BM [day 0] – BM [day i]). **(C)** Representative photographs of the hind paw and digit of DKO* mice (left) and arthritis coring system(right). **(D)** Representative HE staining of digits (38 days post-TAM) **(E)** Quantification of tissue weight at endpoint (38 days post-TAM). Bar graphs and plots represent or include mean ± s.e.m, respectively. Statistical differences between groups at each time point were analyzed by Mann-Whitney test. *P < 0.05, **P < 0.01, ***P < 0.001.

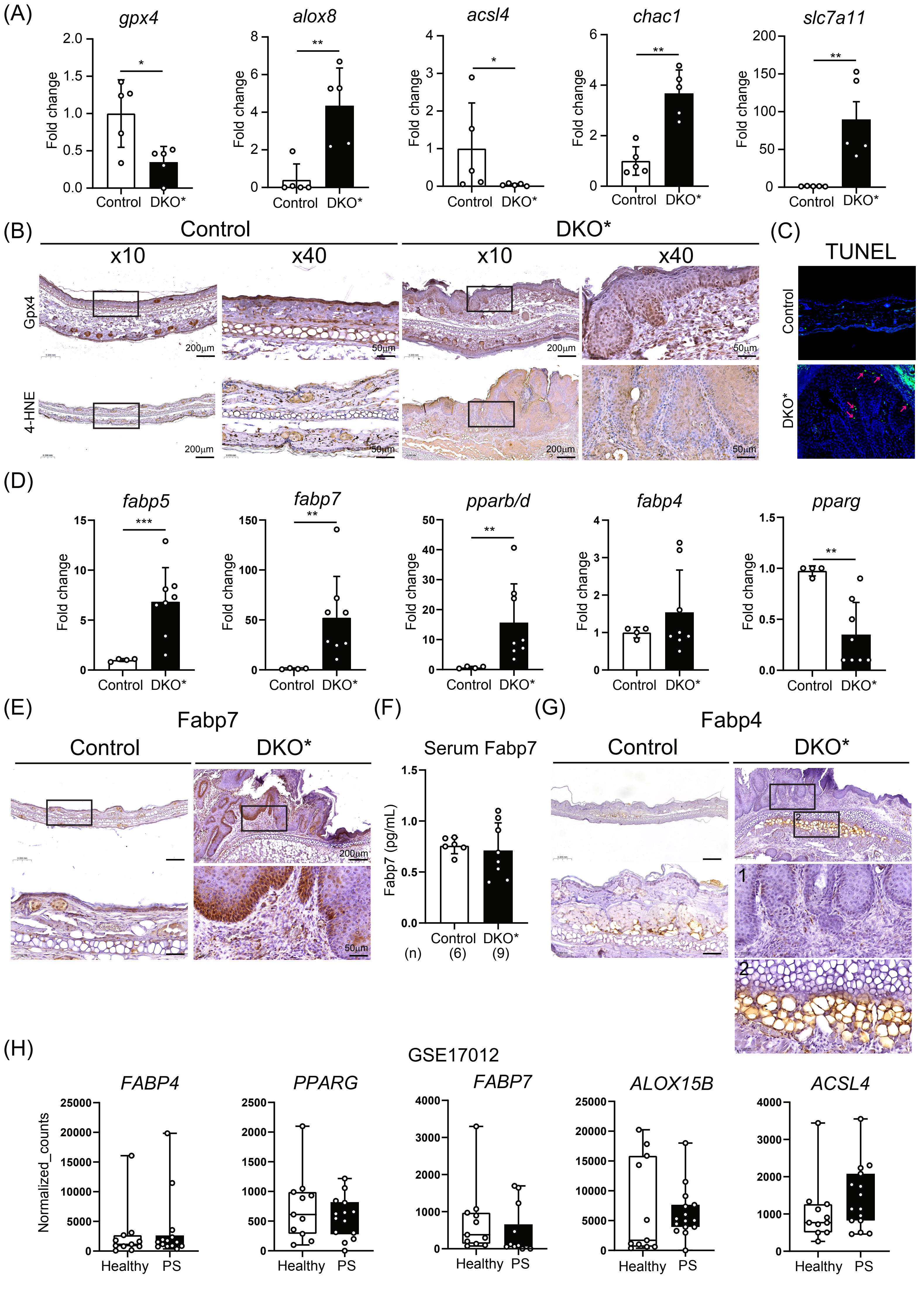
(A)
gpx4
alox8
acsl4
chac1
slc7a11
Fold change
Control
DKO*
(B)
Control
DKO*
x10
x40
Gpx4
4-HNE
200µm
50µm
(C)
TUNEL
Control
DKO*
(D)
fabp5
fabp7
pparb/d
fabp4
pparg
Fold change
Control
DKO*
(E)
Fabp7
Control
DKO*
200µm
50µm
(F)
Serum Fabp7
Fabp7 (pg/mL)
Control
DKO*
(n)
(6)
(9)
(G)
Fabp4
Control
DKO*
1
2
(H)
GSE17012
FABP4
PPARG
FABP7
ALOX15B
ACSL4
Normalized_counts
Healthy
PS

Supplemental Figure 2. Related to Figure 2.

(A) RT-qPCR analysis of ferroptosis-related gene in the ear of control and DKO* mice 14 days post-TAM**. (B)** Representative IHC images of Gpx4 and 4-HNE in control and DKO* ear skin sections 14 days post-TAM injection. **(C)** Terminal deoxynucleotidyl transferase dUTP nick end labeling (TUNEL) staining (green) with DAPI (blue). Red arrows indicate TUNEL positive cells. **(D)** RT-qPCR analysis of *fabp/ppar* genes in the ear of control and DKO* mice 14 days post-TAM. This experimental cohort is independent from the set shown in Figure 2D. **(E)** Representative IHC images of Fabp7 in control and DKO* ear skin sections 14 days post-TAM injection **(F)** Serum Fabp7 in control and DKO* mice 14 days post-TAM injection (ELISA). **(G)** Representative IHC images of Fabp4 in control and DKO* ear skin sections 14 days post-TAM injection. **(H)** Selected transcriptomic changes in whole lesional skin tissue (bulk RNA-seq) from Ps patients compared to healthy individuals (GSE171012, before Secukinumab treatment[35]). Bar graphs and plots represent or include mean ± s.d., respectively. Statistical differences between groups were analyzed by Mann-Whitney test. *$P < 0.05$, **$P < 0.01$, ***$P < 0.001$.

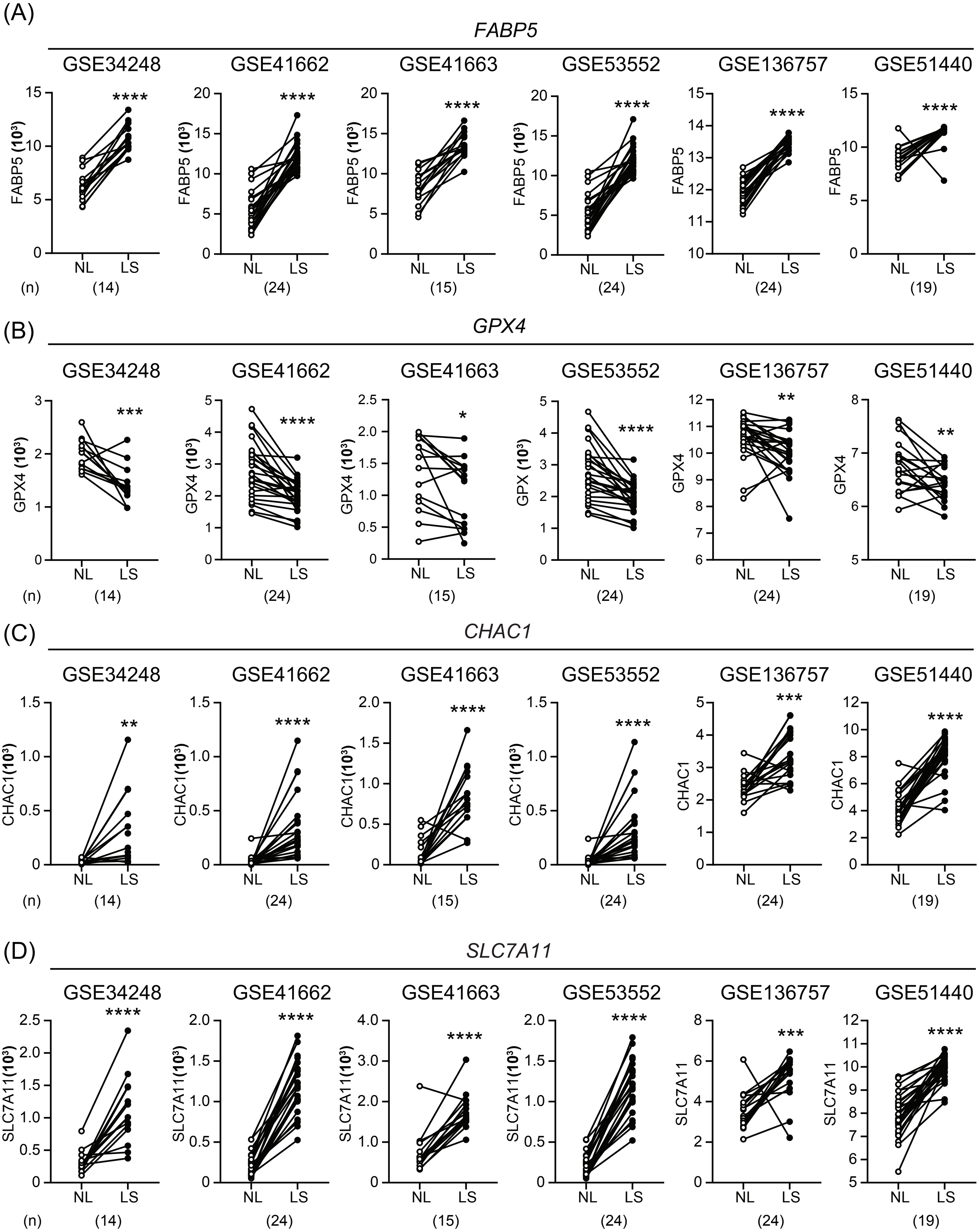
(A)
FABP5
GSE34248
GSE41662
GSE41663
GSE53552
GSE136757
GSE51440
FABP5 (10³)
FABP5
NL
LS
(n)
(14)
(24)
(15)
(24)
(24)
(19)
****
(B)
GPX4
GPX4 (10³)
GPX (10³)
GPX4
***
*
**
(C)
CHAC1
CHAC1(10³)
CHAC1
(D)
SLC7A11
SLC7A11(10³)
SLC7A11

Supplemental Figure 3. Related to Figure 2.

Selected transcriptomic changes comparing non-lesional and lesional skin tissues from Ps patients (GSE34248, GSE41662, GSE41663, GSE53552, GSE136757, and GSE51440). **(A)** FABP5. **(B)** GPX4. **(C)** CHAC1. **(D)** SLC7A11. Statistical differences between non-lesional and lesional areas were analyzed by a paired Wilcoxon signed rank test (non-parametric). *P < 0.05, **P < 0.01, ***P < 0.01 and ***P < 0.0001.

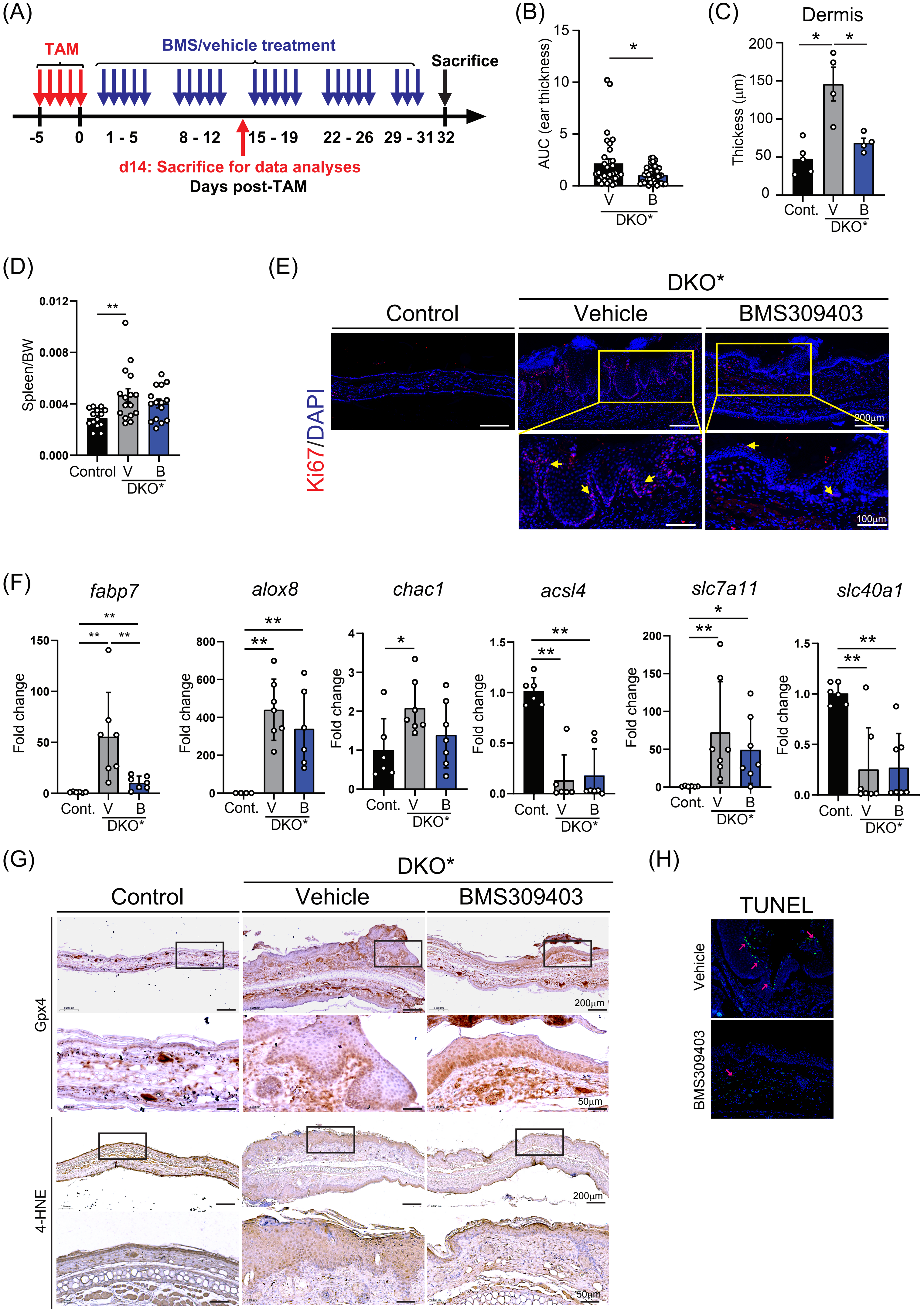

(A)
TAM
BMS/vehicle treatment
Sacrifice
-5
0
1 - 5
8 - 12
15 - 19
22 - 26
29 - 31
32
d14: Sacrifice for data analyses
Days post-TAM
(B)
AUC (ear thickness)
V
B
DKO*
(C)
Dermis
Thickness (μm)
Cont.
V
B
DKO*
(D)
Spleen/BW
Control
V
B
DKO*
(E)
DKO*
Control
Vehicle
BMS309403
Ki67/DAPI
200μm
100μm
(F)
fabp7
alox8
chac1
acsl4
slc7a11
slc40a1
Fold change
Cont.
V
B
DKO*
(G)
DKO*
Control
Vehicle
BMS309403
Gpx4
4-HNE
200μm
50μm
(H)
TUNEL
Vehicle
BMS309403

Supplemental Figure 4. Related to Figure 3

(A) Experimental procedure. Tamoxifen was injected into 8 week-old mice (2mg/mouse/day, 5 consecutive days). BMS309403/vehicle-treatment started one day after the last time TAM injection and lasted for 32 days (5 consecutive days/week). A sub-cohort was sacrificed at day 14 for molecular and histological analyses. **(B)** Quantification of AUC in BMS309403/vehicle-treated DKO* mice between day 0 and day 14. **(C)** Histological quantification of dermal thickness. Bar graphs represent mean ± s.d. **(D)** Quantification of spleen weight 14 days post-TAM injection. **(E)** Representative Ki67 immunofluorescence (IF) images in ear skin sections from control and BMS309403/vehicle-treated DKO* mice 14 days post-TAM injection**. (F)** RT-qPCR analysis of ear skin samples from controls and BMS309403/vehicle-treated DKO* mice 14 days post-TAM injection. **(G)** Representative Gpx4 and 4-HNE IHC images from controls and BMS309403/vehicle-treated DKO* mice 14 days post-TAM injection. Bar graphs and plots represent or include mean ± s.d. Statistical differences between groups were analyzed by Mann-Whitney test. *$P < 0.05$, **$P < 0.01$, and **$P < 0.001$.

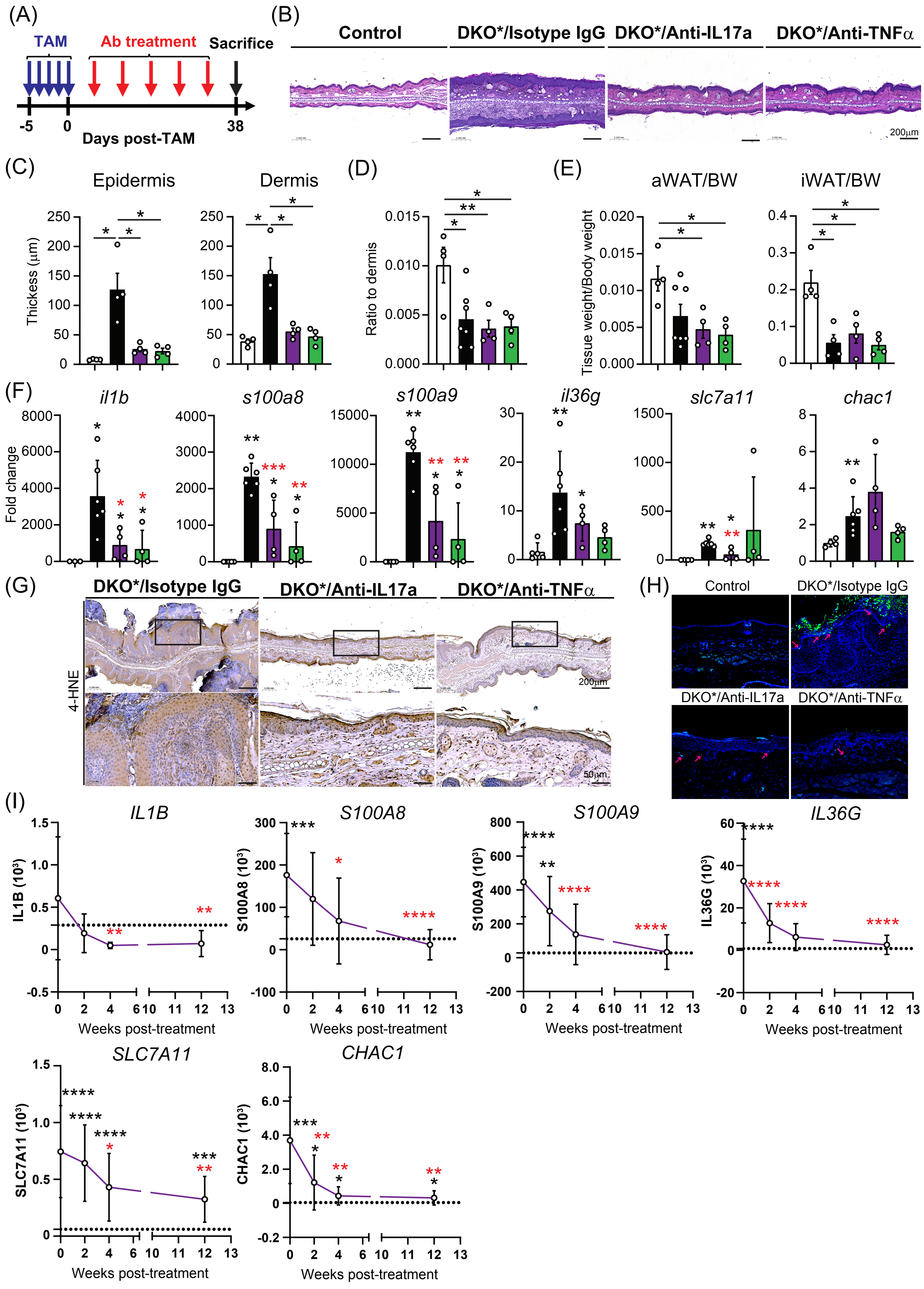

(A)
TAM
Ab treatment
Sacrifice
-5
0
38
Days post-TAM
(B)
Control
DKO*/Isotype IgG
DKO*/Anti-IL17a
DKO*/Anti-TNFα
200μm
(C)
Epidermis
Dermis
Thickess (μm)
(D)
Ratio to dermis
(E)
aWAT/BW
iWAT/BW
Tissue weight/Body weight
(F)
il1b
s100a8
s100a9
il36g
slc7a11
chac1
Fold change
(G)
DKO*/Isotype IgG
DKO*/Anti-IL17a
DKO*/Anti-TNFα
4-HNE
200μm
50μm
(H)
Control
DKO*/Isotype IgG
DKO*/Anti-IL17a
DKO*/Anti-TNFα
(I)
IL1B
S100A8
S100A9
IL36G
SLC7A11
CHAC1
IL1B (10³)
S100A8 (10³)
S100A9 (10³)
IL36G (10³)
SLC7A11 (10³)
CHAC1 (10³)
Weeks post-treatment

Supplemental Figure 5. Related to Figure 4.

(A) Experimental procedure. TAM was injected into 8 week-old mice (2mg/mouse/day, 5 consecutive days). Mice were treated with isotype IgG, anti-IL-17A and anti-Tnfα from 9 weeks of age during 32 days. **(B)** Representative images of H&E staining of ears from DKO* mice treated with anti-IL17a, anti-TNFα, isotype IgG control 38 days post-TAM. **(C)** Histological quantification of epidermal (left) and dermal (right) thickness. **(D)** DWAT area relative to the dermis. **(E)** Quantification of axillary and inguinal adipose tissue weight 32 days post-TAM. **(F)** RT-qPCR analysis of Ps-associated genes using the ear of DKO* mice treated with antibodies 32 days post-TAM (Related to Figure 4D). Black and red asterisks indicate comparisons between untreated control and DKO* mice and between isotype IgG and anti-IL17a/anti-TNFα antibodies, respectively. (C – F) Statistical differences between groups were analyzed by Mann-Whitney test. **(G)** Representative 4-HNE IHC images of ear sections from DKO* mice 38 days post-TAM and the indicated antibody treatments. **(H)** TUNEL staining (green) with DAPI (blue). Red arrows indicate TUNEL positive cells. **(I)** Selected longitudinal transcriptomic changes (bulk RNA-seq) in whole lesional skin tissue from Secukinumab-treated Ps patients compared to healthy individuals (GSE171012). For each gene, the dashed line indicates the expression level measured in healthy individuals from the same dataset. Bar graphs and plots represent or include mean ± s.d., respectively. Statistical differences between groups were analyzed by one-way ANOVA with Tukey's multiple comparisons test. *P < 0.05, **P < 0.01, and ***P < 0.001. Black and red asterisks indicate comparisons between healthy individuals and Ps patients and between week 0 and each time point during secukinumab-treatment, respectively.

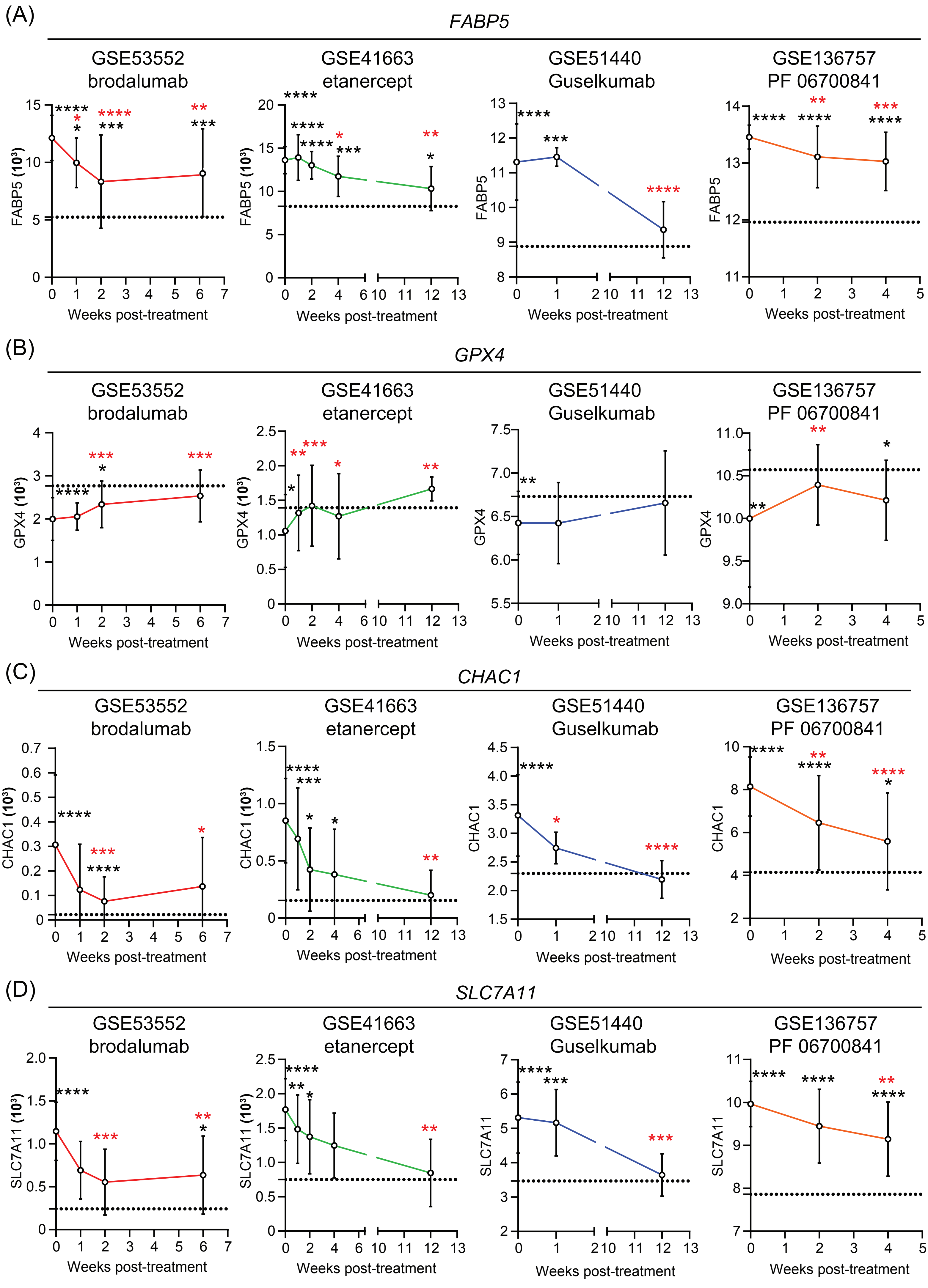
(A)
FABP5
GSE53552 brodalumab
GSE41663 etanercept
GSE51440 Guselkumab
GSE136757 PF 06700841
FABP5 (10³)
FABP5
Weeks post-treatment
(B)
GPX4
GSE53552 brodalumab
GSE41663 etanercept
GSE51440 Guselkumab
GSE136757 PF 06700841
GPX4 (10³)
GPX4
Weeks post-treatment
(C)
CHAC1
GSE53552 brodalumab
GSE41663 etanercept
GSE51440 Guselkumab
GSE136757 PF 06700841
CHAC1 (10³)
CHAC1
Weeks post-treatment
(D)
SLC7A11
GSE53552 brodalumab
GSE41663 etanercept
GSE51440 Guselkumab
GSE136757 PF 06700841
SLC7A11 (10³)
SLC7A11
Weeks post-treatment

Supplemental Figure 6 Related to Figure 4.

Selected longitudinal transcriptomic changes in datasets of whole lesional skin tissue from Ps patients treated with brodalumab (GSE53552), etanercept (GSE41663), guselkumab (GSE51440) and PF06700841 (GSE136757). **(A)** FABP5. **(B)** GPX4. **(C)** CHAC1 **(D)** SLC7A11. Statistical differences between groups were analyzed by one-way ANOVA with Tukey's multiple comparisons test. **P* < 0.05, ***P* < 0.01, and ***P* < 0.01. For each gene, the dashed line indicates the mean expression level measured in the non-lesional skin regions included in each dataset. Bar graphs and plots represent or include mean ± s.d., respectively. Black and red asterisks indicate the comparisons of the lesion area to the non-lesional skin area at each time point and the lesion area at week 0 (pre-treatment) to each time point, respectively. Statistical differences between groups were analyzed by one-way ANOVA with Tukey's multiple comparisons test and/or Mann-Whitney test. **P* < 0.05, ***P* < 0.01, ****P* < 0.01 and ****P* < 0.0001.

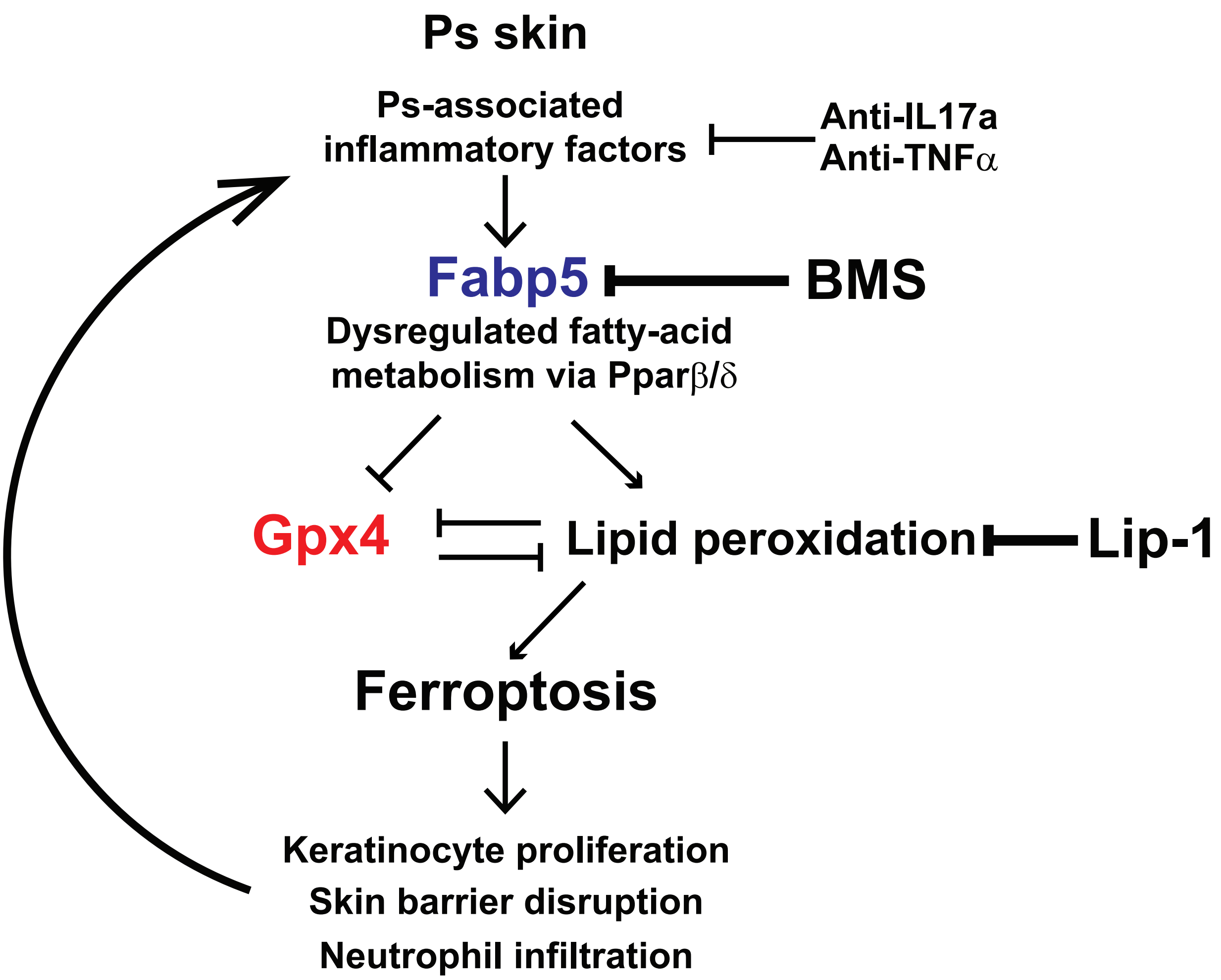
Ps skin
Ps-associated inflammatory factors
Anti-IL17a
Anti-TNFα
Fabp5
BMS
Dysregulated fatty-acid metabolism via Pparβ/δ
Gpx4
Lipid peroxidation
Lip-1
Ferroptosis
Keratinocyte proliferation
Skin barrier disruption
Neutrophil infiltration

Supplemental Figure 7 Scheme depicting amplification of skin inflammation modulated by Fabp5 and Gpx4-mediated ferroptosis.

In the Ps-like skin of DKO* mice, Ps-associated inflammatory factors including IL-23, IL-17, TNF-α promote Fabp5 expression and dysregulate fatty-acid metabolism, where Gpx4-mediated prevention of lipid peroxidation is hampred and subsequently ferroptosis is induced. These events result in keratinocyte proliferation, skin barrier disruption, and neutrophil infiltration, which altogether lead to amplified skin inflammation. Blocking IL-17a, TNF-α and Fabp protein rescues Gpx4 expression, reduces lipid peroxidation and limits skin inflammation.
