# Supplementary material for: Fatty acid-binding protein 5 aggravates psoriasis and psoriasis-like disease through ferroptosis
Source: Cell Death Differ. 2025 Dec 6;33(7):1333–44. doi: 10.1038/s41418-025-01630-4 (PMC13342077; doi:10.1038/s41418-025-01630-4)
Supplement: Supplementary file 2 — Primers [file 41418_2025_1630_MOESM2_ESM.docx]

**Supplemental Table 1. Primers**

**Oligonucleotide primers for quantitative PCR – mouse genes:**

| **Gene** | **Forward primer (5’-3’)** | **Reverse primer (5’-3’)** |
| --- | --- | --- |
| *rlp4* | CTACTGCACTGGCAACCAAA | TCTTGGCAACCACCTTTTTC |
| *fabp2* | GCCTGGACCATTGAGGGAAAT | CTCCTTCATATGTGTAGGTCTGGA |
| *fabp3* | ACCATCATCGAGAAGAACGGG | AGCGTCACCAGTGACTTGAC |
| *fabp4* | TTGGTCACCATCCGGTCAGA | TTCCACCACCAGCTTGTCAC |
| *fabp5* | CGACAGCTGATGGCAGAAAA | CATGACACACTCCACGATCA |
| *fabp6* | CAAGAAGTTCAAGGCTACCGT | CTCCACCAACTTGTCACCCA |
| *fabp7* | GGTTCGGTTGGATGGAGACA | ATCCCCAAAGGTAAGAGTCACG |
| *fabp8* | CATGAAAGCTCTAGGTGTGGGG | GCACTTTCCGTTCTAATGGTGA |
| *il-23* | CACCAGCGGGACATATGAATC | CAGACCTTGGCGGATCCTTT |
| *ltf* | GGCAAGTGCGGTTTAGTTCC | ACAGCAAGGTACCCTTCCAC |
| *slc7a11* | CTGCTCGTAATACGCCCTGG | ATCACCACAGTGATGCCCAC |
| *slc40a1* | TGACAGCTTTGCTGTTGTTGT | GCGTTCTCCCCTTGTTTGTTC |
| *gpx4* | CCGTCTGAGCCGCTTACTT | ATGCACACGAAACCCCTGTA |
| *ftl1* | GCGTCTCCTCGAGTTTCAGA | TCTTGAGATGGCTTCTGCACAT |
| *fth1* | CGGGCTGAATGCAATGGAGT | CAATGAAGTCACATAAGTGGGGA |
| *alox8* | TTTAAAGCCCACTCCGCGTAT | TAGGAACTGGGAGGCGAAGA |
| *chac1* | CTTGAAGACCGTGAGGGCTG | GGTACTTCAGGGCCTCGTTC |
| *cxcl2* | TCATAGCCACTCTCAAGGGC | TCAGGTACGATCCAGGCTTC |
| *il1b* | GCCACCTTTTGACAGTGATGA | AAGGTCCACGGGAAAGACAC |
| *s100a8* | AAATCACCATGCCCTCTACAAG | CCCACTTTTATCACCATCGCAA |
| *s100a9* | ATACTCTAGGAAGGAAGGACACC | TCCATGATGTCATTTATGAGGGC |
| *il36g* | TGATGGCTTTCCCTCCACAA | TGACAGGCTTGACATTGTTGC |
| *nr1c2* | AACATGGAATGTCGGGTGTG | GATCCGATCGCACTTCTCAT |
| *nr1c3* | TTTTCAAGGGTGCCAGTTC | AATCCTTGGCCCTCTGAGAT |
| *cebpa* | GGACAAGAACAGCAACGAGT | CGGTCATTGTCACTGGTCAA |
