# Supplementary material for: Fatty acid-binding protein 5 aggravates psoriasis and psoriasis-like disease through ferroptosis
Source: Cell Death Differ. 2025 Dec 6;33(7):1333–44. doi: 10.1038/s41418-025-01630-4 (PMC13342077; doi:10.1038/s41418-025-01630-4)
Supplement: Supplementary file 3 — Antibody [file 41418_2025_1630_MOESM3_ESM.docx]

**Supplemental Table 2. Antibody**

| **Name** | **Company (catalog no.)** | **Application** | **Conditions** |
| --- | --- | --- | --- |
| 4-HNE | Calbiochem (393207) | WB | 1:3000 dilution |
| GAPDH | HyTest Ltd. (5G4) | WB | 1:5000 dilution |
| Gpx4 | Proteintech (#67763-1) | WB, IHC | 1:1000 (WB) and 1:3000 (IHC) dilution |
| Fabp4 | Abcam (ab92501) | IHC | 1:1000 dilution, Antigen retrieval: Citrate buffer (pH 6.0) in a microwave |
| Fabp5 | Proteintech (12348-1-AP) | IHC | 1:100 dilution, Antigen retrieval: Citrate buffer (pH 6.0) in a microwave |
| Fabp7 | Abcam (ab32423) | IHC | 1:300 dilution, Antigen retrieval: Citrate buffer (pH 6.0) in a microwave |
| Loricrin | Covance (PRB-145P) | IF | 1:2000 dilution, Antigen retrieval: Citrate buffer (pH 6.0) in a pressure cooker |
| Ki-67 | eBioscience (clone SolA15) | IF | 1:1000 dilution, Antigen retrieval: Citrate buffer (pH 6.0) in a pressure cooker |
| Ly6G | Biolegend (Clone 1A8) | IF | 1:500 dilution, Antigen retrieval: Citrate buffer (pH 6.0) in a pressure cooker |
| K5 | Biolegend (Clone Poly9059) | IF | 1:1000 dilution, Antigen retrieval: Citrate buffer (pH 6.0) in a pressure cooker |
| ECL^TM^ donkey anti-rabbit IgG-HRP | Amersham (Clone/Cat. #NA934) | WB | 1:10000 dilution |
| ECL^TM^ sheep anti-mouse IgG-HRP | Amersham (Cat. #NXA931) | WB | 1:10000 dilution |
| Biotinylated Secondary Antibody (Anti-Rabbit) | VECTASTAIN Elite ABC-HRP Kit | IHC | 1:200 dilution, followed by HRP-based chromogen detection (DAB Substrate) kit (Vector laboratories) |
| Biotinylated Secondary Antibody (Anti-Mouse) | VECTOR Laboratories M.O.M. Kit | IHC | 1:250 dilution, followed by HRP-based chromogen detection (DAB Substrate) kit (Vector laboratories) |
| Alexa Fluor 488 goat anti-chicken | Invitrogen (Cat. #A11039) | IF | 1:500 dilution |
| Alexa Fluor 555 donkey anti-rat | Invitrogen (Cat. #A48270) | IF | 1:500 dilution |
| Alexa Fluor 555 donkey anti-rabbit | Invitrogen (Cat. #A31572) | IF | 1:500 dilution |
| anti-mouse TNFα | BioXCell (#BE0058) | in vivo TNFα neutralization | ip, 500μg/injection/mouse |
| anti-mouse IL-17A | BioXCell (#BE0173) | in vivo IL-17A neutralization | ip, 150μg/injection/mouse |
| mouse IgG1 isotype control | BioXCell (#BE0083) | in vivo isotype control | ip, 500μg/injection/mouse |
