# Supplementary figures and images for: Fatty acid-binding protein 5 aggravates psoriasis and psoriasis-like disease through ferroptosis
Source: Cell Death Differ. 2025 Dec 6;33(7):1333–44. doi: 10.1038/s41418-025-01630-4 (PMC13342077; doi:10.1038/s41418-025-01630-4)

Figure 2B

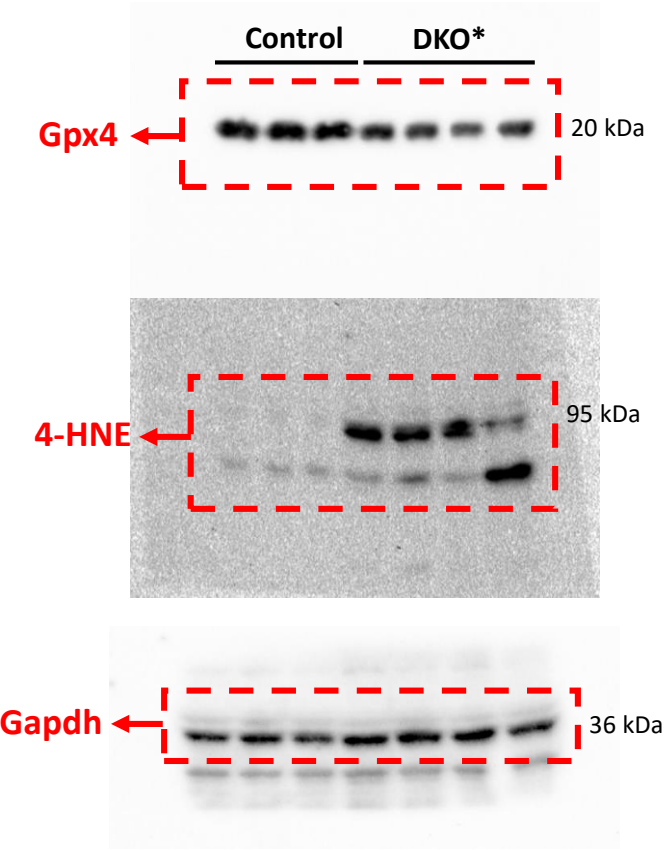

Figure 3L

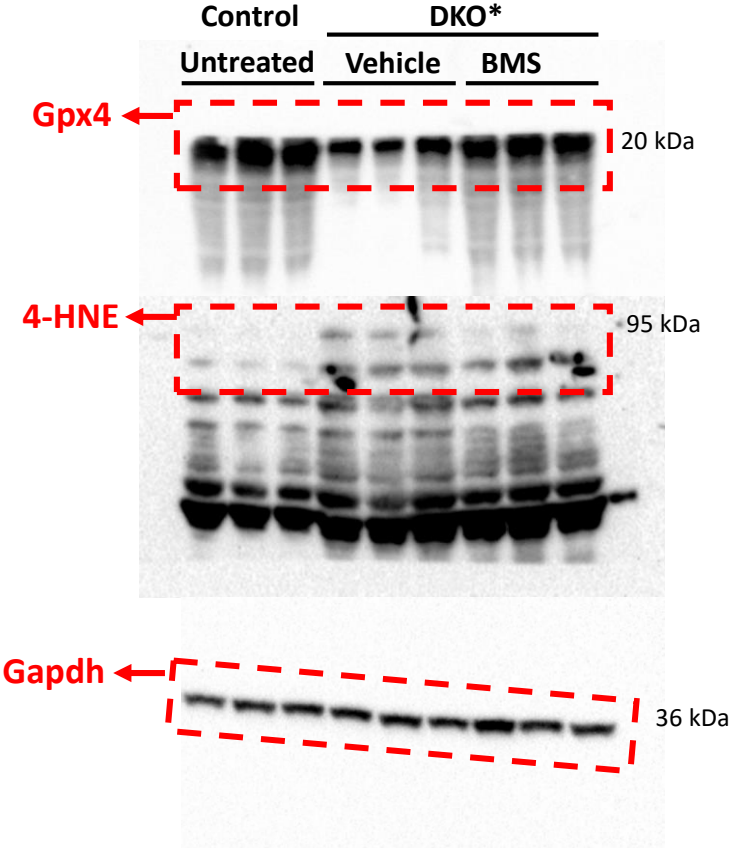

Figure 4F

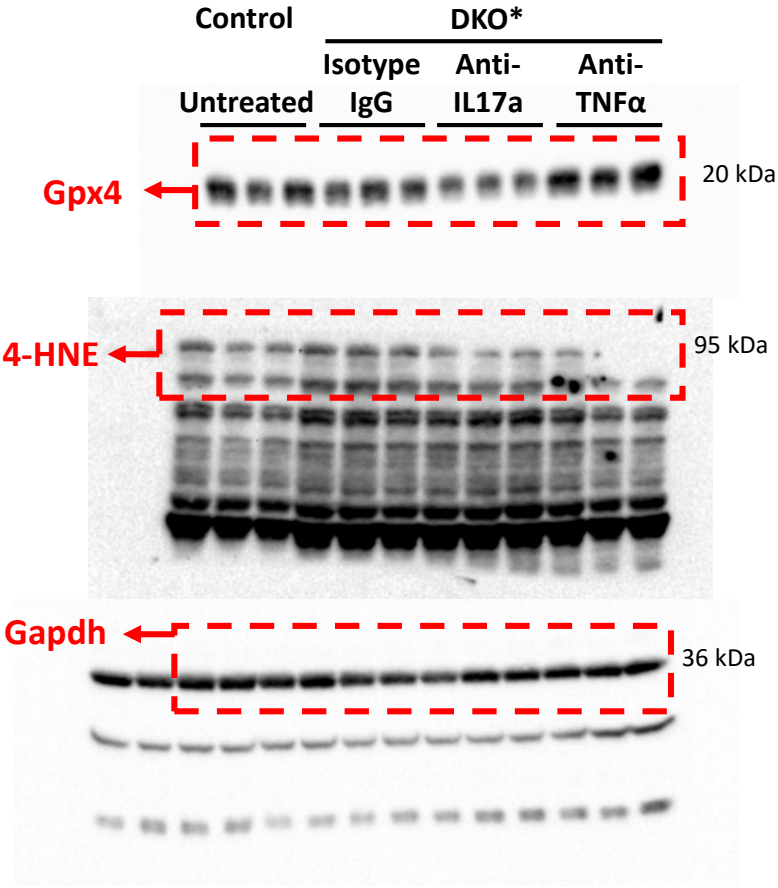

Supplement: Supplementary file 4 — Original Data Files [file 41418_2025_1630_MOESM4_ESM.pdf]
